# Carbohydrate-Binding Non-Peptidic Pradimicins for the Treatment of Acute Sleeping Sickness in Murine Models

**DOI:** 10.1371/journal.ppat.1005851

**Published:** 2016-09-23

**Authors:** Víctor M. Castillo-Acosta, Luis M. Ruiz-Pérez, Juan Etxebarria, Niels C. Reichardt, Miguel Navarro, Yasuhiro Igarashi, Sandra Liekens, Jan Balzarini, Dolores González-Pacanowska

**Affiliations:** 1 Instituto de Parasitología y Biomedicina “López-Neyra”, Consejo Superior de Investigaciones Científicas, Parque Tecnológico de Ciencias de la Salud, Armilla (Granada), Spain; 2 Glycotechnology Laboratory, CIC biomaGUNE, Parque Científico y Tecnológico de Gipuzkoa, San Sebastián, Spain; 3 CIBER of Bioengineering, Biomaterials and Nanomedicine (CIBER-BBN), San Sebastián, Spain; 4 Biotechnology Research Center, Toyama Prefectural University, Imizu, Toyama, Japan; 5 KU Leuven, Rega Institute for Medical Research, Leuven, Belgium; National Institute of Health, UNITED STATES

## Abstract

Current treatments available for African sleeping sickness or human African trypanosomiasis (HAT) are limited, with poor efficacy and unacceptable safety profiles. Here, we report a new approach to address treatment of this disease based on the use of compounds that bind to parasite surface glycans leading to rapid killing of trypanosomes. Pradimicin and its derivatives are non-peptidic carbohydrate-binding agents that adhere to the carbohydrate moiety of the parasite surface glycoproteins inducing parasite lysis *in vitro*. Notably, pradimicin S has good pharmaceutical properties and enables cure of an acute form of the disease in mice. By inducing resistance *in vitro* we have established that the composition of the sugars attached to the variant surface glycoproteins are critical to the mode of action of pradimicins and play an important role in infectivity. The compounds identified represent a novel approach to develop drugs to treat HAT.

## Author Summary

*Trypanosoma brucei*, the causative agent of African trypanosomiasis, is coated with a dense layer of the variant surface glycoprotein (VSG), which plays an essential role in antigenic variation and the ability of the parasite to evade the immune system. VSGs are *N*-glycosylated with complex and/or mannose-rich *N*-glycans by two oligosaccharyltransferase activities in a site-specific manner. Here, we describe that a series of non-peptidic carbohydrate binding agents, previously reported to display antifungal and antiviral properties, exhibit a marked trypanocidal activity. Pradimicin A (PRM-A) and pradimicin S (PRM-S) show specific binding to VSGs and a pronounced cytocidal effect by inducing defects in endocytosis and cytokinesis. Remarkably, PRM-S produces parasitological cure in two different models of acute sleeping sickness. Information on the mode of action was provided by generating PRM-A-resistant parasites, which suffered from defective glycan composition as a consequence of down-regulation of oligosaccharyltransferase genes. Aberrant glycosylation of VSGs resulted in decreased binding of pradimicins but reduced parasite infectivity underscoring the role of glycosylation in virulence. Our findings identify antibiotics with the ability to effectively bind to glycans of the parasite surface as agents capable of affording parasitological cure, thereby providing a novel avenue for design of highly specific drugs to combat African trypanosomiasis.

## Introduction

Human African trypanosomiasis or sleeping sickness is a neglected disease caused by the protozoan parasite *Trypanosoma brucei*. Treatments are largely insufficient and unsatisfactory and new approaches for drug design are highly necessary.

*T*. *brucei* parasites living in the mammalian host rely on antigenic variation to evade the immune system of the host. They are mainly covered by only one kind of a variant surface glycoprotein (VSG) that constitutes an effective barrier that protects from effectors of the host immune system. In the formation of this protective barrier the *N*-glycosylation of VSGs is of major importance [1]. The VSGs are covered by mannose-rich and complex glycans [2–4]. During antigenic variation this shield is changed by expressing new VSGs in a stochastic process known as VSG switching.

Recently, we have reported a series of carbohydrate-binding agents (CBAs) that bind to parasite surface glycoproteins and exhibit a strong trypanocidal activity against the clinically relevant bloodstream form, presenting activity in the nanomolar range [5]. Analysis of the mode of action showed a rapid internalization of glycoprotein-CBA complexes and accumulation in the lysosome leading to perturbation of endocytosis and progression of the cell cycle. Selection for CBA resistance resulted in modification of the *N*-glycan composition of VSGs by changes in the expression of oligosacharyltransferases. Thus, CBAs appear to exert their mode of action against *Trypanosoma* by specifically binding to surface glycans [5, 6].

However, the previously reported antitrypanosomal CBAs were proteins with molecular masses ranging between 8,700 Da (i.e., UDA) and 50,000 Da (i.e., HHA, GNA) or even higher. Proteins present a series of disadvantages to become potential drugs, including efficient scale-up, poor, if any, oral bioavailability and/or potential generation of an immune response.

Non-peptidic, low-molecular-weight antibiotics designated PRM-A and benanomycin A have been discovered in the culture fluid of *Actinomadura hibisca* [7] and *Actinomadura* sp [8], respectively. PRM-A inhibits the growth of fungi (such as *Aspergillus*) [9] and viral infections [10]. It has been shown that PRM-A acts as a lectin in terms of glycan recognition, antiviral activity, and drug resistance patterns [11]. Members of the pradimicin family are unique among natural products in their ability to specifically bind sugars in a Ca^2+^-dependent manner [9]. PRM-S is a highly water-soluble, negatively charged derivative of the antibiotic PRM-A in which the terminal xylose moiety has been replaced by 3-sulfated glucose. The antibiotic is nontoxic against a variety of cell lines, is not mitogenic, and does not induce cytokines or chemokines in peripheral blood mononuclear cell cultures [12]. In addition, pradimicins can be modified chemically, such as BMY28864 which is a derivative of PRM-A. PRM-A and PRM-S exhibit activity against fungi, yeasts and several viruses including HIV and HCV [9, 11–13], while the derivatives PRM-FS [14], PRM-FA-1 [15], BMS181184 [16] and BMY28864 [17] were reported to exhibit efficient antifungal activity.

Here we report that pradimicins inhibit the growth of *T*. *brucei* bloodstream forms at low micromolar concentrations by perturbing cytokinesis and endocytosis and consequently inducing parasite cell lysis. We provide information on their mode of action by generating mutant parasite cells resistant to the drug and examining binding efficiency of the pradimicins to parental and resistant parasitic VSGs and the glycan composition. Furthermore, we found that treatment at 50 mg/kg with PRM-S cures *T*. *brucei brucei* and *T*. *brucei rhodesiense* infection in mice. We propose that pradimicins and carbohydrate-binding agents in general may provide a unique and highly novel avenue for the development of an efficient treatment of parasitic diseases.

## Results

### Pradimicins exhibit *in vitro* trypanocidal activity

**Fig 1:**
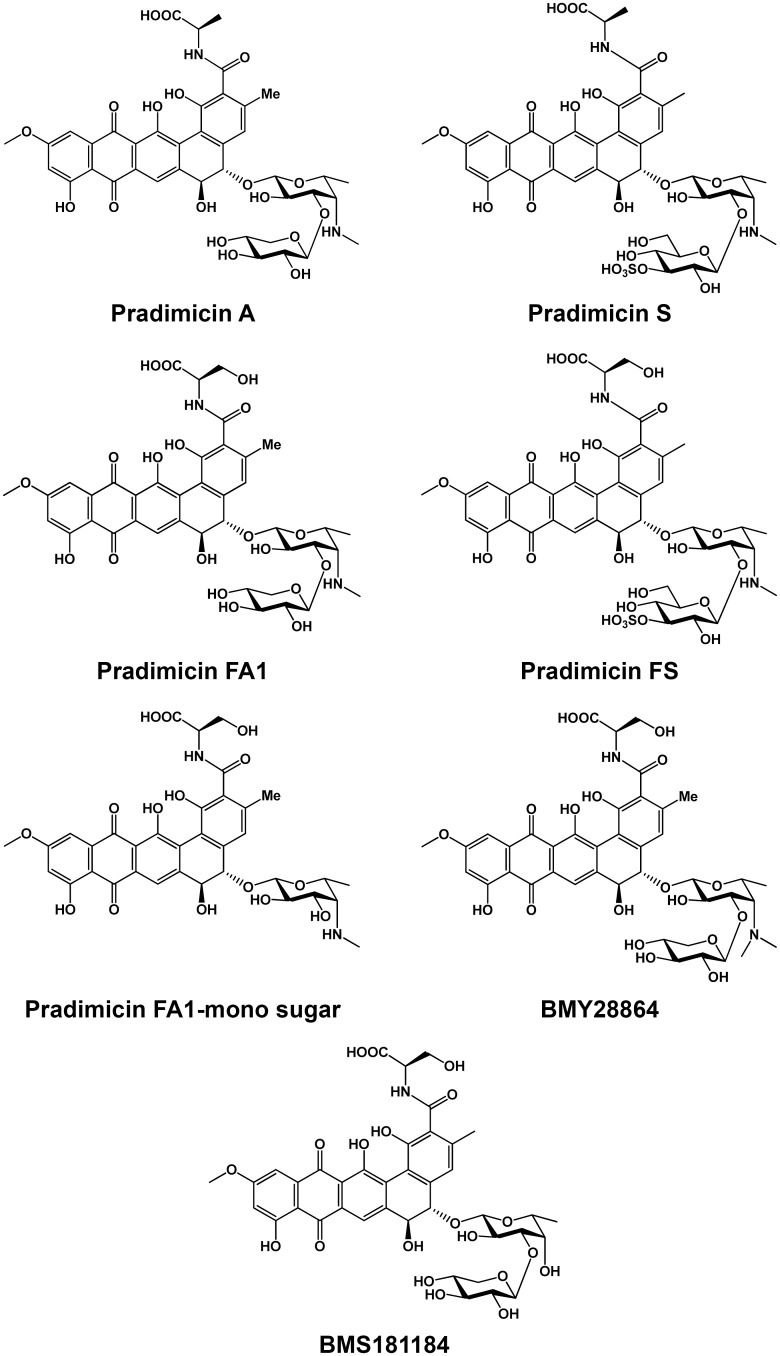
Structural formulae of the pradimicins.

The *in vitro* trypanocidal activities of PRM-A, PRM-S and the derivatives BMY28864, PRM-FS, PRM-FA-1 and BMS181184 were evaluated against the bloodstream forms of *T*. *brucei*. All of them exhibited 50% effective concentration (EC_50_) values in the low micromolar range (Figs 1 and S1, Table 1). PRM-A and PRM-S that preferentially exhibit binding specificity for α(1,2) mannose residues were the most active.

**Table 1:** EC_50_ values of non-peptidic CBAs tested against *T*. *brucei* BSFs.

CBA	Specificity	EC_50_ (μg/ml)	EC_50_ (μM)
PRM-A	α (1,2) Man	3.20 ± 0.04	3.80 ± 0.05
PRM-S	α (1,2) Man	5.85 ± 0.08	6.44 ± 0.08
PRM-FS	D-mannoside	13.0 ± 0.7	13.5 ± 0.8
PRM-FA1-mono sugar	D-mannoside	24.3 ± 2.1	33.3 ± 2.8
BMS181184	D-mannoside	17.1 ± 1.0	20.3 ± 1.2
BMY28864	D-mannoside	18.4 ± 2.4	21.1 ± 2.8

**Fig 2:**
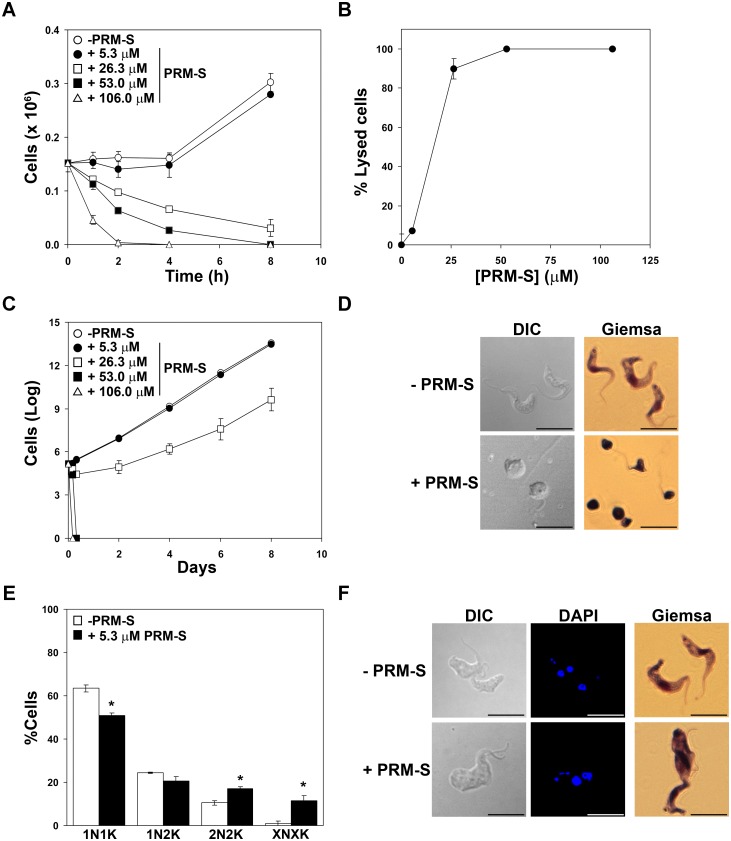
Effect of PRM-S treatment on *in vitro T*. *brucei* BSFs. (A) Plot showing the number of parasites in culture exposed to increasing concentrations of PRM-S (5.3, 26.3, 53.0 and 106.0 μM that correspond to 1-, 5-, 10- and 20-fold the EC_50_, respectively). (B) Plot of the percentage of lysed parasites after PRM-S treatment for 8 h. (C) Accumulated growth after drug removal of parasites treated with PRM-S for 8 h. (D) Microscopy images of cultured samples after exposure to PRM-S for 1 h. Cells were stained with DAPI and Giemsa and observed by fluorescence and light microscopy. (E) Nuclei (N) and kinetoplasts (K) of parasites treated with 5.3 μM for 48 h were stained with DAPI and categorized according to the number of nuclei and kinetoplasts: 1N1K, 1N2K, 2N2K and XNXK. (F) Microscopy images illustrating DIC, DAPI and Giemsa staining of cells exposed to 5.3 μM PRM-S for 48 h. The asterisk shows significant differences calculated by the Student’s *t*-test (n = 2). *, *p* < 0.05. Bars, 10 μm.

A more detailed study of the effect of pradimicins on growth and morphology was accomplished. Thus, a time course of the consequences of exposure to 1-, 5-, 10- and 20-fold the EC_50_ was performed. Parasite viability was severely compromised at the different concentrations tested and total lysis was observed at 10- and 20-fold the EC_50_ after 4 to 8 h of treatment (Fig 2A and 2B). To determine cytocidal activity, PRM-S was removed after 8 h of exposure at different concentrations and growth was monitored thereafter. PRM-S behaved as a trypanocidal agent at concentrations 10-fold the EC_50_ since complete abolishment of growth was achieved at this and higher concentrations (Fig 2C). Furthermore, after 1 h of incubation with PRM-S at 53.0 μM, cells exhibited a rounded shape and detachment of the flagellum (Fig 2D). In order to identify cell cycle alterations, the distribution of nuclei and kinetoplasts by DAPI staining was examined after PRM-S exposure at 5.3 μM (EC_50_) for 48 h (Fig 2E and 2F). The microscopic analysis revealed a slight increase of cells which have completed mitosis (2N2K) (17%), as well as the emergence of a population with multiple nuclei and kinetoplasts (XNXK) (11.5%) suggesting that PRM-S impairs cytokinesis by binding to the variant surface glycans.

### PRM-S binds to the VSGs of the trypanosome surface leading to endocytosis defects

**Fig 3:**
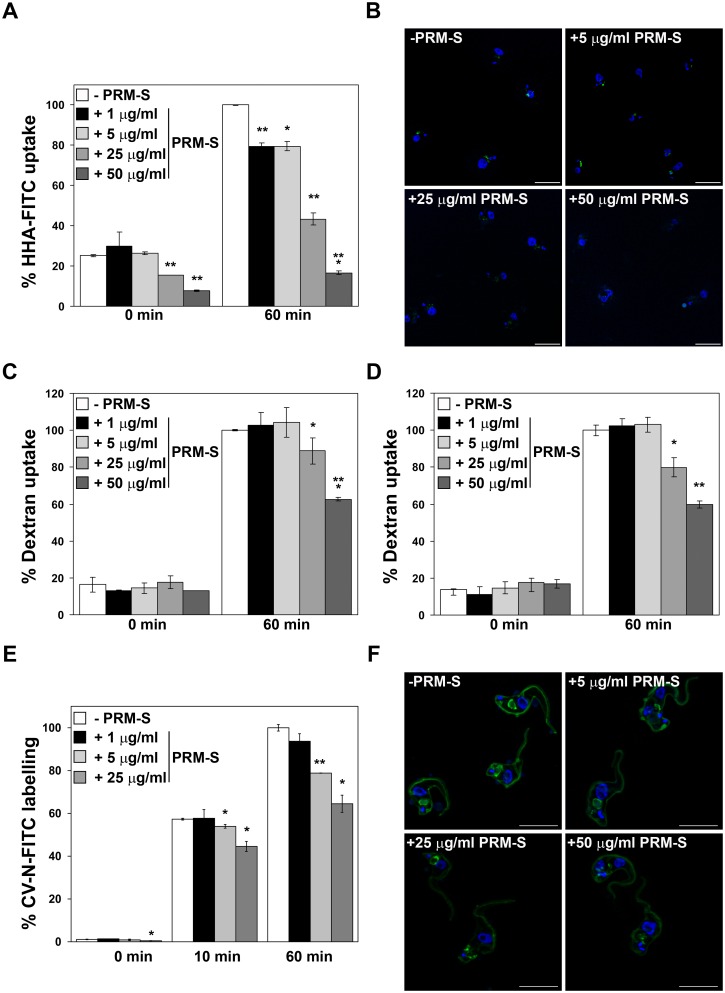
PRM-S binding and fluid-phase endocytosis analysis. PRM-S binding to parasite surface VSGs was evaluated by competition labelling assays using HHA, a lectin that binds to VSGs and is rapidly endocytosed [5] or CV-N, a lectin that binds to the surface coat of the trypanosomes. Labelling was measured by FACS at 0 min, 10 min and 60 min of incubation at increasing PRM-S concentrations and visualized by 3D microscopy. (A and B) Quantification (A) and images (B) of the labelling with HHA-FITC (1 μg/ml) in the presence of PRM-S. (C and D) Fluid-phase endocytosis analysis using Alexa Fluor 488-labelled dextran 10,000 in the absence (C) or in the presence (D) of HHA (1 μg/ml). (E and F) Quantification (E) and images (F) of the labelling with CV-N-FITC (0.6 μg/ml) in the presence of PRM-S. Bars, 10 μm. The asterisks show significant differences calculated by the Student’s *t*-test (n = 3). *, *p* < 0.05, **, *p* < 0.005 and ***, *p* < 0.0005 vs the parental strain.

As a first approach for assessing effective pradimicin binding to glycans of the surface glycoproteins, competition assays were performed between PRM-S and HHA, a lectin that has been previously reported to bind VSGs [5], and PRM-S and CV-N, a lectin with a similar α(1,2) Man specificity as PRM-S. Accordingly, different PRM-S concentrations were examined first in the presence of HHA-FITC conjugates and fluorescence was analysed at 0 min and 60 min of incubation by flow cytometry and microscopy. HHA binding to the surface coat (determined at 0 min) was significantly decreased by 1.7 and 3-fold after incubation with 25 μg/ml and 50 μg/ml PRM-S, respectively (Fig 3A) and uptake was drastically reduced at the highest concentration tested compared to the control without PRM-S (Fig 3A and 3B). We also monitored fluid-phase endocytosis (dextran uptake) in the presence and absence of PRM-S and HHA in order to discard possible effects of HHA on endocytosis that could be interpreted as a decrease in binding/uptake of PRM-S. Fig 3C shows that while PRM-S produces a significant reduction in dextran internalization at 25 to 50 μg/ml in the absence of additional HHA, HHA at 1 μg/ml does not affect endocytosis at the PRM-S concentrations tested (Fig 3D). In the case of competition assays with CV-N-FITC conjugates, fluorescence was analysed at 0, 10 and 60 min of incubation by flow cytometry and microscopy. The presence of 25 μg/ml PRM-S reduced CV-N binding to the surface coat at 0 and 60 min of incubation to 59% and 64% of the control values respectively (Fig 3E and 3F). Therefore, we conclude by these observations that PRM-S binds to glycans of the trypanosome surface glycoproteins, such as the VSGs, in a similar fashion to HHA and CV-N and subsequently, the PRM-S-VSGs complex triggers endocytosis defects and parasite death.

### Selection *in vitro* of PRM-A-resistant parasites

PRM-A-resistant parasites were generated in order to provide an insight into the mode of action. Adaption of the parasites to this CBA was achieved by exposure to stepwise increasing concentrations of PRM-A and cell lines named PRM-A25, PRM-A50 and PRM-A100 were generated that grew and were isolated at concentrations of 25, 50 and 100 μg/ml PRM-A, respectively (corresponding to 8-, 16- and 32-fold the EC_50_ value for the parental cell line). The resistance selectivity index (ratio EC_50_ resistant parasite/EC_50_ parental parasite) was determined as an estimate of the degree of drug resistance. A value of 25 was obtained for PRM-A25 and PRM-A50, and of 40 for PRM-A100 (Table 2). The drug-resistance phenotype was retained after 3 months in the absence of PRM-A pressure suggesting that we are dealing with a stable and genetically-encoded phenotype.

**Table 2:** EC_50_ values for parental (BSF) and PRM-A-resistant *T*. *brucei* cell lines. Resistance indices (R-index) for PRM-A-resistant cells with respect to the parental strain are indicated.

Cell line	EC_50_ (μg/ml)	EC_50_ (μM)	R-index^a^
*Tb* BSF	3.2 ± 0.1	3.8 ± 0.05	1.0
PRM-A25	82 ± 4	98 ± 4	25.8
PRM-A50	86 ± 2	103 ± 3	27.1
PRM-A100	127 ± 7	152 ± 8	40.0
PRM-A50 p-Rem (3M)^b^	95 ± 12	113 ± 15	29.7
PRM-A100 p-Rem (3M)^b^	126 ± 5	150 ± 6	39.6

^a^Resistance selectivity index or ratio EC_50_ PRM-A-resistant cells/EC_50_ parental cells.

^b^Post-removal time period of cell culture in the absence of PRM-A is indicated between parentheses.

Cross-resistance was analysed for the PRM-A100 cell line using a variety of CBAs with predominant different specificities; HHA (α(1,3)-α(1,6) Man), EHA (Man), GNA (α(1,3) Man), NPA (α(1,6) Man), UDA (GlcNAc oligomers) and PRM-S (α(1,2) Man) were tested. Cross-resistance indices (R-index) were calculated as ratios of EC_50_ PRM-A100 /EC_50_ parental cell line (BSF). PRM-A100 cells exhibited significant resistance to PRM-S, HHA and EHA and low resistance to UDA, NPA and GNA (Table 3).

**Table 3:** EC_50_ values and cross-resistance (R) indices of PRM-A100 and parental cell lines.

CBAs	Specificity	EC_50_ *Tb* BSF (μM)	EC_50_ PRM-A100 (μM)	R-index^a^
HHA	α(1,3)- α(1,6) Man	0.020 ± 0.001	0.23 ± 0.03	11.5
EHA	Man	0.014 ± 0.0003	0.17 ± 0.02	11.9
GNA	α(1,3) Man	0.043 ± 0.002	0.19 ± 0.01	4.5
NPA	α(1,6) Man	0.058 ± 0.001	0.29 ± 0.008	5.0
UDA	GlcNAc oligomers	0.225 ± 0.006	1.7 ± 0.002	7.4
PRM-S	α(1,2) Man	6.4 ± 0.1	77.7 ± 0.8	12.1

^a^Resistance selectivity index or ratio EC_50_ PRM-A100-resistant cells/EC_50_ parental cells.

### PRM-A exposure to *T*. *brucei* causes changes in VSG *N*-glycosylation

**Fig 4:**
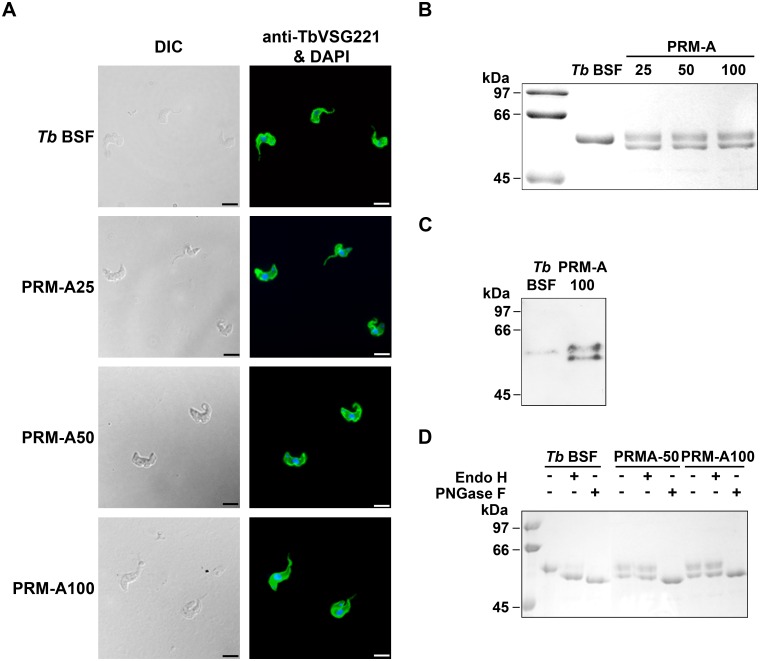
Analysis of the nature and *N*-glycosylation status of the VSG expressed in PRM-A-resistant trypanosomes. (A) Indirect immunofluorescence microscopy analysis of VSG221 expression in parasites resistant to PRM-A probed with a polyclonal antibody against VSG221. Nuclear and kinetoplast DNA was stained with DAPI. Bars, 10 μm. (B) sVSGs of parental and resistant strains were purified as described [20] and analysed by SDS/PAGE and Coomassie blue staining. (C) Western-blot analysis of sVSG isolated from parental and PRM-A100 cells using an anti-TbVSG221 polyclonal antibody. (D) Endoglycosidase treatment of sVSG samples with Endo H (that removes oligomannose *N*-linked glycans) or PNGase F (that removes all *N*-linked glycans), followed by SDS/PAGE and Coomassie blue staining analysis.

We have reported previously that prolonged exposure to peptidic CBAs lead to induction of CBA resistance caused by genotypic changes in the *N*-glycosylation profile [5, 6]. In order to evaluate whether the non-peptidic CBAs would produce a similar phenotype, the expression of VSGs and their *N*-glycosylation nature were analysed in parasites resistant to PRM-A. First, indirect immunofluorescence using an anti-TbVSG221 polyclonal antibody [18] revealed that VSG221 expression was maintained in all PRM-A-resistant cells (Fig 4A). Secondly, the soluble form of VSG (sVSG) was isolated and resolved using SDS/PAGE and Coomassie Blue staining. Unlike parental cells, sVSGs of all PRM-A-resistant cells appeared as doublets, which were positively identified as VSG221 by tryptic peptide mass fingerprinting using MALDI-TOF analysis (Voyager DE PRO, AB Sciex). The existence of two forms of sVSG with different migration properties (Fig 4B and 4C), in addition to differences in endoglycosidase digestion (Fig 4D), clearly indicated the induction of modifications in the *N*-glycan nature of the resistant parasite strains. Endo H or PNGase F, which remove conventional triantennary oligomannose and hybrid *N*-glycans or all types of *N*-glycans respectively, were used to confirm this. Parental sVSG harbours an Endo H-resistant (Asn263) and an Endo H-sensitive (Asn428) *N-*glycosylation site, coming from the action of different oligosaccharyltransferase activities, such as TbSTT3A and TbSTT3B which transfer Man_5_GlcNAc_2_ or mainly Man_9_GlcNAc_2_ structures, respectively [19]. In the case of PRM-A-resistant strains, after Endo H treatment no molecular mass shift was observed, while PNGase F digestion converted both bands into a fully deglycosylated form (Fig 4D). Therefore, two glycoforms of VSG221 seem to coexist in the resistant population. The VSG221 sequences of PRM-A50 and PRM-A100 strains were identical to that of the parental *T*. *brucei* BSF strain used in this study ruling out the possibility that changes in glycosylation are due to mutations in the *N*-glycosylation sites (S2 Fig).

**Fig 5:**
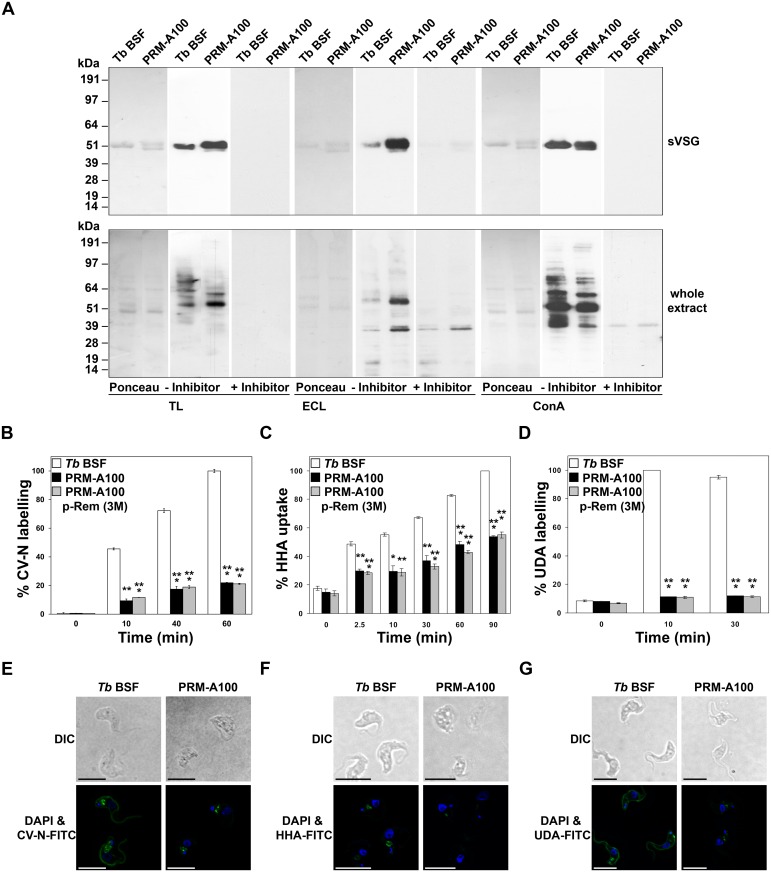
Assessment of the VSG glycosylation status by lectin blotting and labelling with lectins. (A) sVSG and cell pellets lysates obtained after hypotonic lysis from parental and PRM-A100 lines were subjected to SDS-PAGE, transferred to a nitrocellulose membrane and probed with TL, ECL and ConA lectins. Ponceau staining was used as loading control. Addition of chitin hydrolysate (inhibitor), D-lactose and α-methyl mannose was used as a specificity control for TL, ECL or ConA, respectively. (B-G) Live cells of parental and PRM-A100 lines were labelled with CV-N-FITC (B and E), HHA-FITC (C and F) or UDA-FITC (D and G) conjugates and fluorescence was quantified by FACS analysis and visualized by 3D microscopy. Bars, 10 μm. The asterisks show significant differences calculated by the Student’s *t*-test (n = 3). *, *p* < 0.05, **, *p* < 0.01 and ***, *p* < 0.005 vs the parental strain.

Blotting with lectins of different specificities was performed to further characterize the nature of the *N*-glycans. We used the TL lectin that recognizes poly-*N*-acetyl lactosamine [21] or the Manβ1-4GlcNAcβ1-4GlcNAc trisaccharide core of paucimannose glycans [22], ECL displaying preference for single LacNAc units [23] but also with any poly-*N*-acetyl lactosamine-containing glycoproteins, and ConA with oligomannose- and hybrid glycans-containing Manα1-3(Manα1–6)Manα1-specificity [24–26]. In addition, chitin hydrolysate, D-lactose, and α-methylmannose, which are inhibitors of TL, ECL and ConA, respectively, were included in the study as specificity controls. Fig 5A shows that the binding of TL or ECL to sVSGs was significantly increased in PRM-A100, which further suggests changes in the glycosylation status from oligomannose to paucimannose *N*-glycans containing mainly one or several *N*-acetyl lactosamine structures. However, the additional presence of any abbreviated core structures derived from paucimannose *N*-glycans cannot be discarded. In contrast, the binding of ConA to sVSG was unaltered in the PRM-A-resistant cells. When whole cell extracts were probed with TL, ECL and ConA, moderate changes in the glycosylation patterns were observed.

Changes in *N*-glycosylation were also assessed by labelling with lectin-FITC conjugates with different glycan binding specificities: CV-N-FITC (α(1,2) Man), HHA-FITC (α(1,3)- α(1,6) Man) and UDA-FITC (GlcNAc oligomers). Thus, resistant cells had a strongly reduced ability to bind to all of the lectins tested, CV-N (Fig 5B and 5E), HHA (Fig 5C and 5F) and UDA (Fig 5D and 5G), even after culture for 3 months in the absence of PRM-A.

For a more detailed view of *N*-glycosylation of VSG221 from PRM-A100-resistant parasites, free and procainamide glycans after enzymatic cleavage with PNGase F were analysed by both MALDI-TOF MS and (ultra)-high performance liquid chromatography-fluorescence coupled to mass spectrometry (UPLC-FLD/MS). Assignment of peaks was based on exact mass and previous biochemical knowledge of the trypanosome glycome or via diagnostic fragment ions [19]. The two major peaks of MALDI-TOF spectra obtained for *Tb* BSF free glycans at *m/z* 932.877 and 1905.687, were assigned to paucimannose Man_3_GlcNAc_2_ (H3N2 with *m/z* 932.877) and triantennary oligomannose Man_9_GlcNAc_2_ (H9N2, *m/z* 1905.687), respectively. In line with previously published trypanosoma VSG glycosylation profiles, further peaks were tentatively assigned to the oligomannoses structures such as the biantennary H4N2 (Man_4_GlcNAc_2_, *m/z* 1095.025) or the triantennary H5N2 (Man_5_GlcNAc_2_, *m/z* 1257.171), H6N2 (Man_6_GlcNAc_2_, *m/z* 1419.308), H7N2 (Man_7_GlcNAc_2_, *m/z* 1581.433) and H8N2 (Man_8_GlcNAc_2_, *m/z* 1743.564). In addition, we observed peaks corresponding to the biantennary hybrid *N*-glycans H3N3 (Man_3_GlcNAc_3_, *m/z* 1136.077), H4N3 (Man_4_GlcNAc_3_, *m/z* 1298.221), H5N3 (Man_5_GlcNAc_3_, *m/z* 1460.358) and H6N3 (GlcMan_5_GlcNAc_3_, *m/z* 1622.476). Further peaks in the glycan profile were assigned to the poly-*N*-acetyl lactosamine complex *N*-glycans Gal_2_Man_3_GlcNAc_4_ (H5N4, *m/z* 1663.538) and Gal_4_Man_3_GlcNAc_6_ (H7N6, *m/z* 2394.116) (S3 Fig). The analysis of glycan composition mostly agrees with the results previously obtained by Manthri *et al* for VSG221 [19].

**Fig 6:**
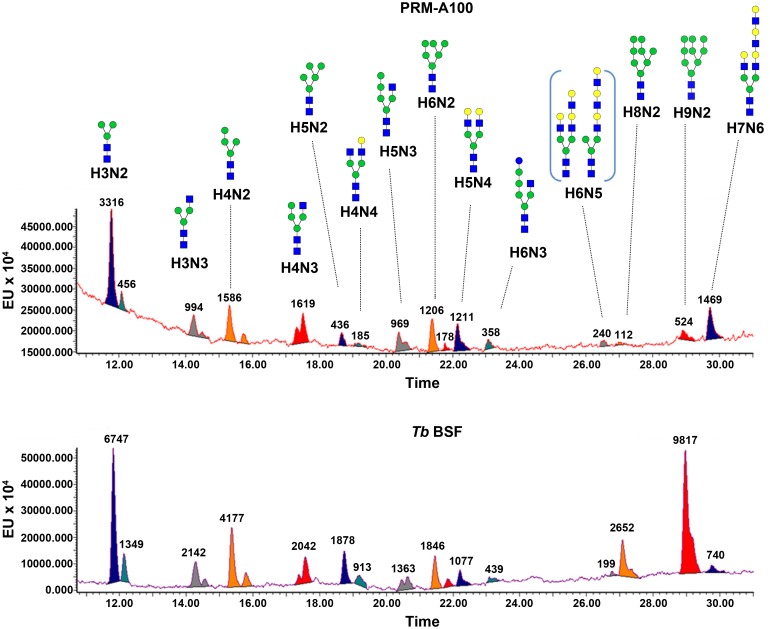
Analysis of *N*-glycans from PRM-A100-resistant sVSG221. Integrated UPLC-FLD chromatograms of procainamide labelled glycans from resistant (PRM-A100) and control (*Tb* BSF) *N*-glycan samples labelled with procainamide.

In the PRM-A100 resistant cell line, the oligomannose structures H3N2, H4N2, H5N2, H6N2 and H9N2 were found whereas H7N2 and H8N2 were absent. In addition, peaks corresponding to hybrid *N*-glycans (H3N3, H4N3, H5N5 and H6N3) and complex *N*-glycans (H5N4 and H7N6) were identified. The two new H4N4 (*m/z* 1501.401) and H6N5 (*m/z* 2028.827) species present in the profile were assigned to GalMan_3_GlcNAc_4_ and Gal_3_Man_3_GlcNAc_5_ structures, respectively (S4 Fig). In-source fragmentation of ions at *m/z* 1663.538, 2028.827 and 2394.116 produced diagnostic fragments indicating the presence of poly-*N*-acetyl lactosamine structures in the H6N5 and H7N6 species and terminal *N*-acetyl lactosamine in the H5N4 species (S5–S7 Figs). UPLC-FLD/MS analysis provided a more quantitative view of the glycan distribution present on *Tb* BSF VSG221 highlighting H9N2 triantennary oligomannose and H4N2 and H3N2 paucimannose structures as major compounds together with minor amounts of other triantennary oligomannose, hybrid and complex *N*-glycans. In contrast, UPLC-FLD/MS analysis of procainamide labelled glycans from sVSG221 of PRM-A100-resistant parasites presented a ~8-fold decrease in oligomannose structures (H9N2 and H8N2) and a significant increase in the hybrid (H4N3, H5N3 and H6N3) and complex *N*-glycans with poly-*N*-acetyl lactosamines (H5N4, H6N5 and H7N6), whereas paucimannoside levels (H3N2, H4N2 and H3N3) remained unaltered (Fig 6 and Table 4). Therefore, these results firmly demonstrate that alterations in *N*-glycosylation occur in response to PRM-A pressure, leading to an *N*-glycan profile with a lower content of oligomannose structures containing α(1,2) or α(1,3)-α(1,6) bonds and the emergence of complex glycans with terminal poly-*N*-acetyl lactosamine motifs, which would be responsible for the decreased binding affinity of pradimicins.

**Table 4:** Relative quantification (% abundance) of *N*-glycans from control (*Tb* BSF) and resistant (PRM-A100) cell lines. Data extracted and normalized from UPLC-FLD chromatograms.

Mannosides	*Tb* BSF (%)	PRM-A 100 (%)	Hybrid *N*-glycans	*Tb* BSF (%)	PRM-A 100 (%)	Complex *N*-glycans	*Tb* BSF (%)	PRM-A 100 (%)
H3N2	17.8	22.4	H3N3	5.7	1.2	H4N4	2.4	1.2
H4N2	11.0	10.7	H4N3	5.4	10.9	H5N4	2.8	8.2
H5N2	5.0	2.9	H5N3	3.6	6.5	H6N5	0.5	1.6
H6N2	4.9	8.1	H6N3	1.2	2.4	H7N6	2.0	9.9
H8N2	7.0	0.8						
H9N2	25.9	3.5						

### Pradimicins interact directly with parasite VSG221

**Fig 7:**
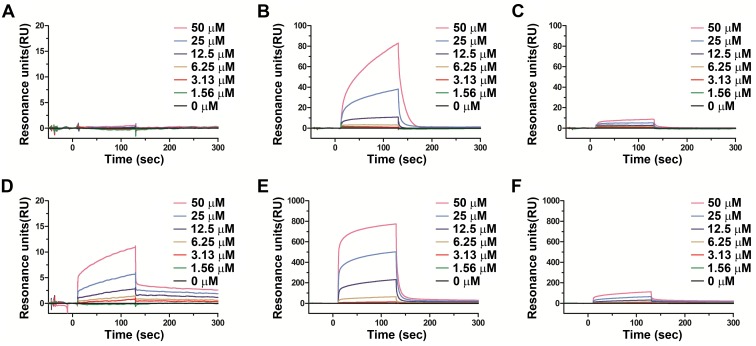
Surface plasmon resonance analysis. (A, B and C) SPR analysis of serial dilutions of PRM-A from 1.56 to 50 μM exposed to the parental *Tb* BSF VSG221 bound at low density (826 RU) (A) or high density (6,500 RU) (B) and to the PRM-A100-resistant cell line VSG221 bound at high density (6,070 RU) (C) on a CM5 sensor chip. (D, E and F) SPR analysis of serial dilutions of PRM-S using the same conditions as described above.

To confirm that indeed pradimicin interacts with parasite-encoded VSGs, surface plasmon resonance studies (SPR) were performed using VSG221 derived from parental (BSF) and drug (PRM-A100)-resistant *T*. *brucei* cell lines. Both VSGs were immobilized on a sensorchip. Parental *Tb* BSF VSG221 was bound at low and high density (826 and 6,500 RU, respectively) and PRM-A100 VSG221 derived from the drug-resistant parasites at high density (6,070 RU) (Fig 7). PRM-A binding to the low-density wild-type *Tb* BSF VSG221-based sensorchip was hardly visible in the sensorgrams. Only 50 μM PRM-A provided a poor binding amplitude (Fig 7A). Instead, using the high-density parental *Tb* BSF VSG221 sensorchip, concentration-dependent binding of PRM-A could be observed (Fig 7B). Interestingly, binding of PRM-A to the high-density PRM-A100-resistant VSG221-bound sensorchip caused also a concentration-dependent binding amplitude, but at a ~ 10-fold lower efficiency than to the parental *Tb* BSF VSG221 that was immobilised at comparable densities (6,070 and 6,500 RU, respectively) (Fig 7C). A similar phenomenon was observed for the more-soluble PRM-S derivative (Fig 7D–7F). A significant concentration-dependent binding to the parental *Tb* BSF VSG221-bound sensorchip was observed and this binding was much more pronounced for the high-density compared to the low-density *Tb* BSF VSG221 sensorchip. As demonstrated for PRM-A, binding of PRM-S to PRM-A-resistant VSG221 was 6- to 7-fold less pronounced than to the parental VSG221.

Attempts to calculate the binding affinities (K_D_) of the pradimicins, as previously reported for HIV gp120 [12], to parental and PRM-A-resistant VSG221, including determination of the k_on_ and k_off_ rates failed, mainly due to the lack of 1:1 stoichiometric kinetics of the binding. Indeed, given the small size of the pradimicins, it might be assumed that several pradimicin molecules can bind on one single VSG molecule given the high amount of glycans present on VSG. In addition, the pradimicin antibiotics are known to internally staple (associate) at higher (micromolar) concentrations, further compromising relevant calculations of the K_D_ values for the parental and resistant VSGs. Nevertheless, the SPR-based binding study of the pradimicins to parental and PRM-A-resistant VSG convincingly revealed that PRM-A and PRM-S concentration-dependently bind to VSG and that both PRM-A and PRM-S showed a compromised binding efficiency for the PRM-A100-resistant *versus* the parental *Tb* BSF VSG. These findings confirm the specific binding of the pradimicins to parasitic VSGs, and the poorer binding of PRM-A and PRM-S to PRM-A-resistant VSG than parental VSG. In addition, they support our view and provide further evidence that resistance against pradimicins is due to the glycan changes in the parasitic VSG.

### Prolonged PRM-A exposure induces down-regulation of the *STT3A* and *STT3B* genes yielding parasites with reduced infectivity in mice

**Fig 8:**
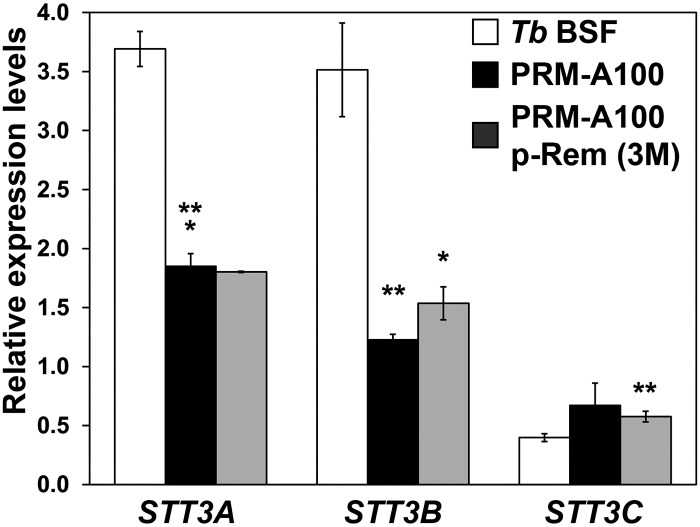
Relative expression of *TbSTT3* genes in PRM-A-resistant trypanosomes. The mRNA levels of the *STT3* genes in the PRM-A100 cell line cultured in the presence or absence of PRM-A and those of the parental line were determined by RT-qPCR and normalized with regard to the expression of the *myosin 1B*. Values were calculated from triplicates of three independent experiments. The asterisks show significant differences calculated by the Student’s *t*-test (n = 3). *, *p* < 0.005, **, *p* < 0.001 and ***, *p* < 0.0001 vs the parental strain.

Obvious candidates potentially involved in the changes in carbohydrate composition are the oligosaccharyltransferase (OST) activities coded by three genes: STT3A, STT3B and STT3C [5, 6]. OSTs mediate *N*-glycosylation of VSGs in a site specific manner [19, 27, 28]. Accordingly, *TbSTT3A*, *TbSTT3B* and *TbSTT3C* mRNA levels were examined by RT-qPCR in the PRM-A100 cell line. Specific primers designed against the variable region of each gene were used in the analysis [5]. The occurrence of recombination events between these genes was also examined using a combination of primers. S7 Fig shows that mutant parasites harbour only canonical genes. However a reduction in the expression levels of *TbSTT3A* (2.0-fold) and *TbSTT3B* (2.9-fold) in PRM-A-resistant cells compared to the parental line was observed, even when PRM-A pressure was removed for up to 3 months (Fig 8). In order to identify modifications in the nucleotide sequence that could be responsible of the changes in mRNA levels, the sequences of the *TbSTT3* gene open reading frames as well as their corresponding 5’UTRs and 3’UTRs were determined as described in supporting information (S8 Fig and S1 Text). Three nucleotide changes were found in the *TbSTT3A* coding sequence with regard to the database sequence resulting in the amino acid changes E510G, K513E and L705P (S1 Table). In the case of TbSTT3B, a G248S replacement was identified while no differences were found in *TbSTT3C*. Further analysis is currently underway to ascertain the functional significance of the mutations affecting the *TbSTT3* genes. No differences were observed in the sequences of the STT3 UTRs.

**Fig 9:**
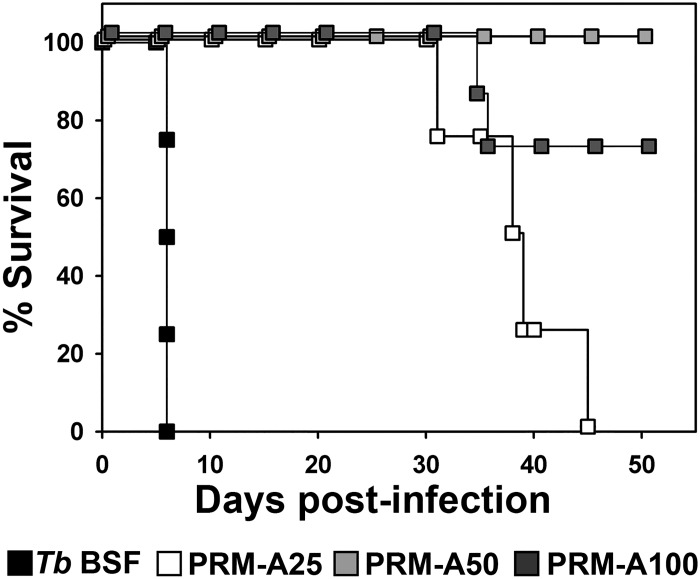
Survival analysis of mice infected with PRM-A-resistant trypanosomes. Kaplan-Meier survival analysis of mice infected with parental (*Tb* BSF) and PRM-A25, PRM-A50 and PRM-A100-resistant parasites.

Parasites from the parental, PRM-A25, PRM-A50 and PRM-A100-resistant cell lines were used to infect mice and survival was monitored. We observed a strong parasite fitness cost for the three resistant cell lines that resulted in reduced infectivity. Whereas the parental line exhibited a median survival days (MSD) of 6.0 ± 0.0 days, the four mice infected with PRM-A25-resistant parasites and two mice out of the seven infected with the PRM-A100-resistant parasites died showing an MSD of 38 ± 7 days and 34.5 ± 0.7 days, respectively. All the mice infected with PRM-A50 were alive after 50 days (MSD >50 days) (Fig 9). Thus, the PRM-A-resistant parasites invariably showed a pronounced compromised infectivity potential in mice.

### TbSTT3B is involved in resistance to pradimicins

**Fig 10:**
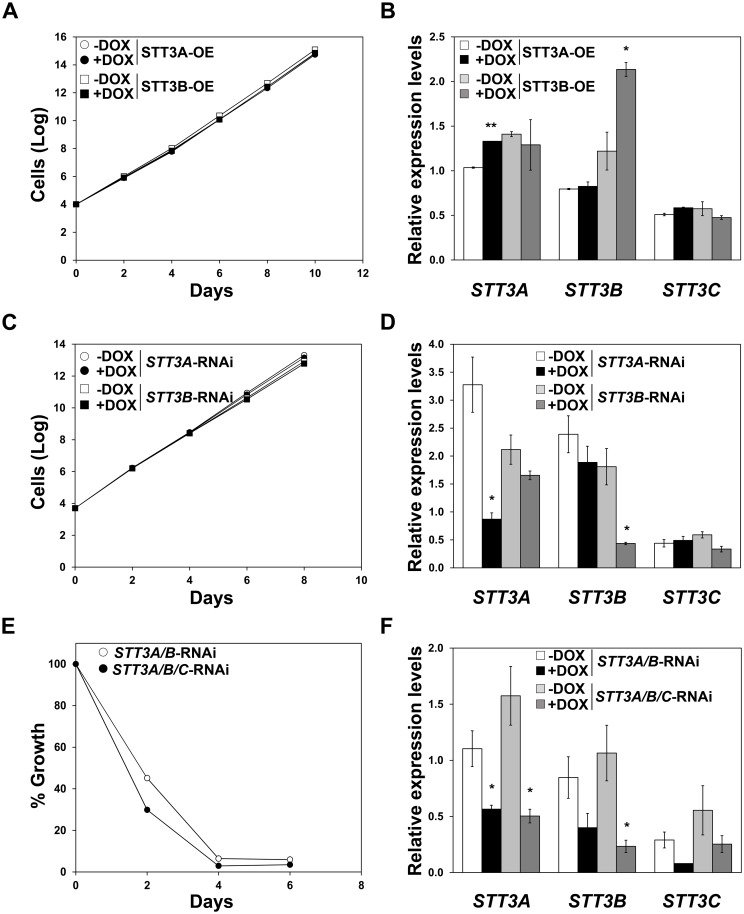
Effect of overexpression or RNAi mediated depletion of STT3A, STT3B and STT3C on resistance to PRM-S. (A and B) Accumulated growth (A) and mRNA levels (B) of PRM-A100 cells overexpressing TbSTT3A or TbSTT3B. (C and D) Accumulated growth (C) and mRNA levels (D) of *STT3A*-RNAi and *STT3B*-RNAi parental cells. (E and F) Growth profile (E) and mRNA levels (F) of *STT3A/B*-RNAi and *STT3A/B/C*-RNAi parental cells. -DOX, non-induced; +DOX, cells exposed to doxycycline (1 μg/ml). Relative expression of the *STT3* mRNA levels was determined by RT-qPCR and normalized with regard to the expression of *myosin 1B*. Values were calculated from triplicates of two independent experiments. The asterisks show significant differences between induced *versus* corresponding non-induced cell lines calculated by the Student’s *t*-test (n = 2): *, *p* < 0.05 and **, *p* < 0.001.

With the aim of establishing if defective expression of OSTs is the major factor involved in resistance to pradimicins, overexpression of TbSTT3A and TbSTT3B in the PRM-A100 cell line and conversely RNAi mediated depletion of *TbSTT3A*, *TbSTT3B* and *TbSTT3C* in the parental line were accomplished (S1 Text). Firstly, PRM-A100 cells were transfected individually with constructs that allowed for the expression of *TbSTT3A* or *TbSTT3B*, yielding the PRM-A100 STT3A-OE and PRM-A100 STT3B-OE cell lines, respectively (Fig 10A). The mRNA levels of the *STT3* genes were evaluated by RT-qPCR. *TbSTT3B* mRNA increased significantly after induction in the PRM-A100 STT3B-OE cell line, while *TbSTT3A* mRNA levels were maximally 1.3-fold enhanced upon induction of PRM-A100 STT3A-OE (Fig 10B). The determination of EC_50_ values established that sensitivity to PRM-S in the PRM-A100 STT3B-OE parasites increased upon induction 12.1-fold with regard to the parent PRM-A100 strain (EC_50_ 77.7 ± 0.8 μM) thus pointing towards a major role for this OST in the resistance mechanism. Moderate overexpression of TbSTT3A did not result in sensitization to PRM-S, thus curtailing its role in the resistance phenotype (Table 5). On the other hand, RNAi-mediated depletion of *TbSTT3* genes was evaluated individually or simultaneously in the wild-type strain. Thus, the cell lines *Tb* BSF *STT3A*-RNAi, *Tb* BSF *STT3B*-RNAi, *Tb* BSF *STT3A/B*-RNAi and *Tb* BSF *STT3A/B/C*-RNAi were generated. While the knockdown of TbSTT3A or TbSTT3B [5] had no effect on growth, simultaneous knockdown of TbSTT3A and TbSTT3B or TbSTT3A, TbSTT3B and TbSTT3C resulted in severe growth defects (Fig 10C and 10E), in agreement with previous studies showing that *N*-glycosylation is essential [28] (Fig 10D and 10F). Depletion of TbSTT3B gave rise to high resistance to PRM-S (14.3-fold) whereas RNAi-mediated reduction of TbSTT3A had no notable consequences and even slightly sensitizes parasites to the drug (0.6-fold) compared to the parental line (EC_50_ 5.3 ± 0.2 μM) (Table 5). These results confirm the central role of OSTs in defining the VSG glycosylation profile and PRM-S binding capacity. Specifically TbSTT3B, which transfers Man_9_GlcNAc_2_ rendering oligomannose *N*-glycans, appears as the main player in the molecular mechanism responsible for resistance to pradimicins.

**Table 5:** EC_50_ values for PRM-S of PRM-A100 parasites overexpressing TbSTT3A or TbSTT3B, and wild type parasites subjected to RNAi mediated depletion of TbSTT3A, TbSTT3B and TbSTT3C.

Cell line	EC_50_ non-induced cells (-DOX) (μM)	EC_50_ induced cells (+DOX) (μM)
**PRM-A100 STT3A-OE**	88.5 ± 6.4	69.7 ± 2.0
**PRM-A100 STT3B-OE**	71.2 ± 5.9	6.4 ± 1.1
***Tb* BSF STT3A-RNAi**	7.4 ± 0.2	3.4 ± 0.1
***Tb* BSF STT3B-RNAi**	6.75 ± 0.08	75.9 ± 0.2

### Resistance to PRM-A results in altered endocytosis

**Fig 11:**
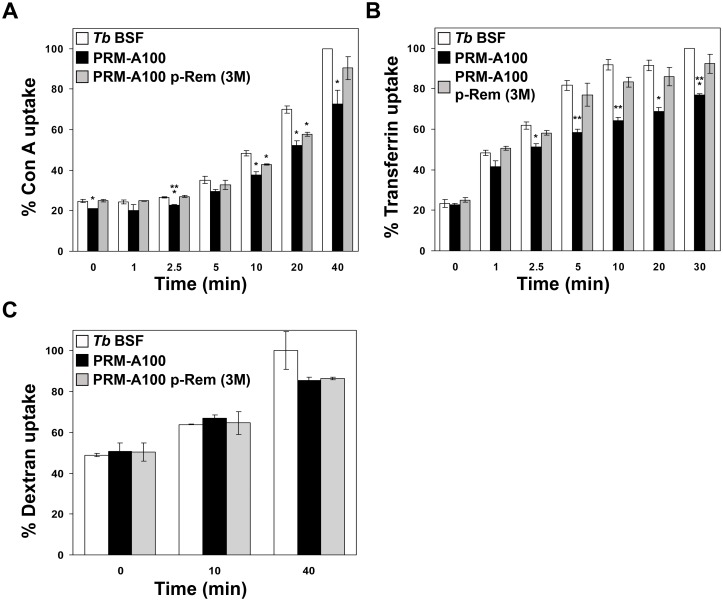
Endocytosis analysis. PRM-A100 cells grown both in the presence and absence of PRM-A were used to evaluate receptor-mediated, receptor independent and fluid-phase endocytosis. The uptake of ConA (A), transferrin (B) as well as Alexa Fluor 594-dextran 10,000 (C) conjugates were measured and compared with uptake in the parental line. The asterisks show significant differences calculated by the Student’s *t*-test (n = 3). *, *p* < 0.05, **, *p* < 0.01 and ***, *p* < 0.005 vs the parental strain.

To evaluate whether PRM-A resistance involved modifications in endocytosis, PRM-A100-resistant and parental parasite strains were probed with ConA as a marker for membrane-bound endocytic activity [29], transferrin as a receptor-mediated endocytosis marker, and dextran as a fluid-phase endocytosis marker. PRM-A-resistant parasites exhibit a slightly reduced capacity to internalize both ConA and transferrin, although internalization was restored when trypanosomes were cultured in the absence of PRM-A and remain resistant (Fig 11A and 11B). On the other hand no differences were found in dextran uptake between resistant and parental parasites (Fig 11C). Whereas ConA interiorization is dependent on the interaction with surface glycans, transferrin and dextran uptake (fluid phase endocytosis) are mostly independent of protein glycosylation. Indeed mutant non-glycosylated ESAG6 and ESAG7 (the two subunits forming the transferrin receptor) are capable of forming a heterodimer and of binding transferrin [30]. Nonetheless, changes in the glycan nature of the transferrin receptor could interfere with transferring binding due to steric hindrance. We conclude that the minor reversible defects observed in ConA and transferrin uptake do not have a major role in the resistance phenotype.

### *In vivo* PRM-S treatment produces parasitological cure in murine models of acute sleeping sickness

**Fig 12:**
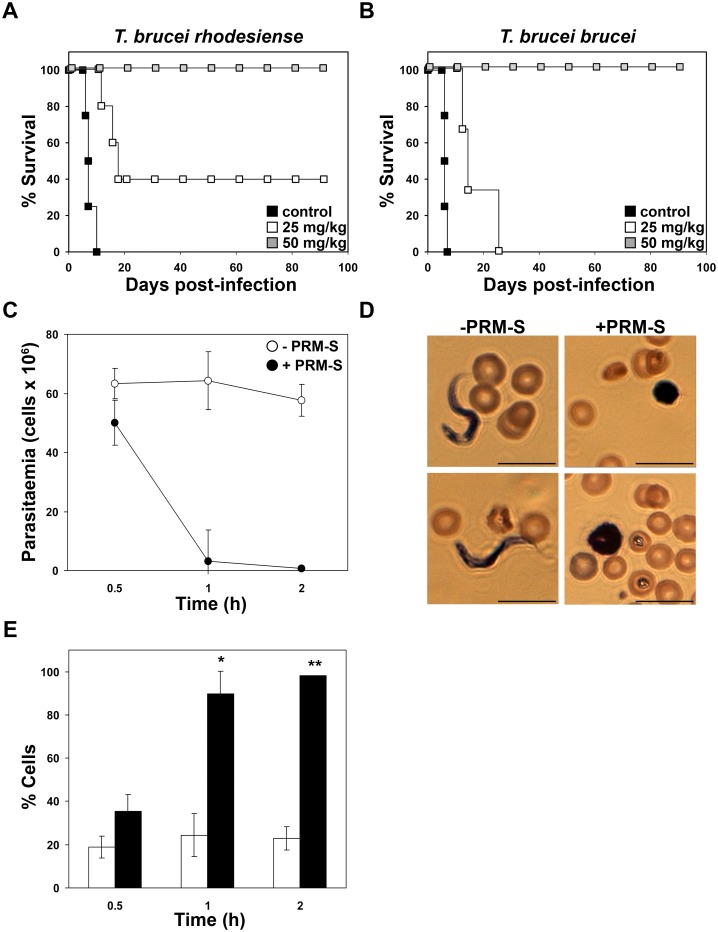
Treatment of *T*. *brucei*- infected mice with PRM-S. (A and B) Kaplan-Meier survival analysis of mice infected with *T*. *brucei rhodesiense* EATRO3 ETat1.2 TREU164 (A) or with *T*. *brucei brucei* single-marker strain 427 (B) and treated with 25 mg/kg, and 50 mg/kg of PRM-S as well as with the vehicle used as control. (C) Parasitaemia course of mice infected with *T*. *brucei brucei* single-marker strain 427 at short periods after PRM-S administration (50 mg/kg). (D) Light microscopy of cells stained with Giemsa from mice infected with *T*. *brucei brucei* at 1 h after PRM-S administration. (E) Quantification of the percentage of cells exhibiting a rounded shape, which was observed by Giemsa staining. The asterisk shows significant differences calculated by the Student’s *t*-test (n = 2). *, *p* < 0.05, and **, *p* < 0.005. Bars, 10 μm.

Given the limited solubility and availability of PRM-A, the trypanocidal effect of several PRM-A derivatives, including PRM-S, BMS181184 and BMY28864, was examined in mice using an acute model of African trypanosomiasis. Mice were infected with the *T*. *brucei rhodesiense* EATRO3 ETat1.2 TREU164 or *T*. *brucei brucei* single-marker 427 strains. PRM-S exhibited a dosage-related efficacy at intraperitoneal dosages of 25 mg/kg and 50 mg/kg per day administered on four consecutive days. At 25 mg/kg PRM-S, two of five mice infected with *T*. *brucei rhodesiense* were cured (Fig 12A), and the survival markedly improved for the treated animals since the MSD was 14.0 ± 3.0 days and the mean relapse days (MRD) 10.7 ± 0.6 days, while controls treated only with the drug vehicle formulation exhibited a MSD of 7.5 ± 1.7 days (Table 6). In the case of *T*. *brucei brucei-*infected mice, at 25 mg/kg all the mice died although the MSD was extended to 18.0 ± 7.0 days with a MRD of 10.7 ± 0.6 days with regard to an MSD of 6.2 ± 0.5 days in the control (non-treated) group (Fig 12B and Table 6). Notably, at 50 mg/kg, PRM-S produced parasitological cure of all the mice infected with either *T*. *brucei rhodesiense* or *T*. *brucei brucei* (Fig 12A and 12B).

**Table 6:** *In vivo* activities of pradimicin derivatives against *T*. *brucei*.

Parasites	Mice	Drug	dose mg/kg days 3–6	route	cured/ infected	MSD	MRD
*T*. *brucei rhodesiense* EATRO3	Balb/C	-		i.p.	0/4	7.5 ± 1.7	
		PRM-S	4 x 25	i.p.	2/5	14.0 ± 3.0	10.7 ± 0.6
			4 x 50	i.p.	5/5	>90	
		-		i.p.	0/4	6.2 ± 0.5	
		BMS181184	3 x 50	i.p.	0/4	12.8 ± 3.1	9.0 ± 2.2
		BMY28864	3 x 50	i.p.	0/4	6.2 ± 0.5	
*T*. *brucei brucei* 427 SM	C57BL/6J	-		i.p.	0/4	6.2 ± 0.5	
		PRM-S	4 x 25	i.p.	0/3	18.0 ± 7.0	10.7 ± 0.6
			4 x 50	i.p.	6/6	>90	

Parasites from *T*. *brucei brucei-*infected mice treated with 25 mg/kg PRM-S were isolated just before animal sacrifice, inoculated into HMI-9 medium and exposed to increasing concentration of PRM-S for determination of the EC_50_ value. The EC_50_ obtained (5.0 ± 0.2 μM) was similar to the control, showing that short term PRM-S exposure *in vivo* did not generate resistance to PRM-S.

The parasitaemia and morphology were determined in mice infected with *T*. *brucei brucei* at 30 min, 1 h and 2 h after drug treatment (50 mg/kg) in order to provide an insight into the mechanism of action *in vivo*. PRM-S provokes a rapid parasite clearance (Fig 12C) and a pronounced increase in the population of cells with a rounded shape (90%) (Fig 12D and 12E) suggesting that direct interaction with the parasite surface glycans is the mode of action in the mouse model.

Other pradimicin derivatives somewhat less active *in vitro* than PRM-A/PRM-S were also investigated *in vivo*. Specifically, BMS181184 and BMY28864 at a single dosage of 50 mg/kg were used to treat mice infected with *T*. *brucei rhodesiense*. BMS181184 produced parasite clearance after the first dosage yet further relapsed (MRD of 9.0 ± 2.2 days), while the MSD was extended to 12.8 ± 3.1 days, thereby doubling the survival of the control group (MSD of 6.25 ± 0.5 days). BMY28864-treated animals died at the same time as the control group, and therefore, proved not to be active *in vivo* (S9 Fig and Table 6).

## Discussion

In this study we have explored the trypanocidal activity of pradimicins and the mode of action of these non-peptidic CBAs in order to provide an insight into the potential of these highly novel antiparasitics. Pradimicins are low-molecular-weight antibiotics (~ 900 Da) that exhibit antiviral and antifungal properties mediated by lectin-mimic binding to surface glycans [11, 12, 14, 17, 31]. We show that these CBAs exhibit a remarkable trypanocidal activity *in vitro* in the low micromolar range, in particular PRM-A and its highly water-soluble derivative PRM-S proved most active. Extraordinarily, PRM-S also exhibits a potent trypanocidal effect *in vivo*, resulting in a parasitological cure in acute models of African trypanosomiasis using both the *T*. *brucei rhodesiense* and *T*. *brucei brucei* species. These findings are a continuation of previous work conducted in our laboratory where we identified a series of plant lectins such as HHA, UDA, GNA, NPA and EHA, that exhibit strong inhibitory activity against *T*. *brucei* [5]. Our observations were in contrast to the general belief that most lectins are not toxic for *T*. *brucei* bloodstream forms since rapid internalization and degradation of the surface glycoprotein-lectin complex would result in a lack of toxicity [32]. Although the dissociation constant of the PRM-A-VSG221 or PRM-S-VSG221 complexes could not be determined in detail due to the existence of multiple binding sites in the VSG molecule, we provide multiple evidence that the mode of action of pradimicins is indeed due to tight binding to surface VSGs and perturbation of the endocytic pathway resulting in a rapid parasite death. Defects in endocytosis of a similar fashion have been observed earlier upon formation of VSG-specific nanobody complexes (Nsbs) and have been reported to play an essential role in the nanobody’s cytotoxic action [33].

Studies on the molecular mechanisms of resistance to the pradimicins were designed in order to shed light on the mode of action of these compounds. Thus resistance was generated by a step-wise selection to PRM-A and a high resistance index was achieved for mutant cells that also exhibited cross-resistance to PRM-S and to other mannose-binding lectins. The resulting resistance phenotype was characterized by defects in the *N*-glycosylation pathway that resulted in an altered *N*-glycosylation of VSGs and other glycoproteins which presumably lead to a reduced binding of the CBA. Indeed in the resistant mutant parasites, lectin blotting analysis together with the observation that CV-N, HHA and UDA uptake and binding are impaired suggested profound modifications in surface glycans. Given that this phenotype remained after withdrawal of drug pressure, we concluded that the resistance phenotype was genetically encoded and stable. On the other hand, defects in endocytosis in the resistant mutants were minor and reversible upon drug withdrawal. Transferrin and ConA uptake reduction was reversed after culture in the absence of PRM-A while no defects were observed in fluid-phase endocytosis thereby establishing that this process is not relevant to the resistance phenotype.

Pradimicins and benanomicins comprise a unique family of antibiotics with a lectin-like ability to bind D-mannose (D-Man) in the presence of Ca^2+^ [8, 9, 34]. Lately they have been attracting attention as the only class of non-peptidic small molecules that can capture D-Man under physiologically relevant conditions [35]. The evidence available suggests that PRM-A recognizes the 2-, 3-, and 4-hydroxyl groups of D-Man although binding to pyranosides of l-Fuc and l-Gal when the Ca^2+^ concentration is not excessive has also been reported [35]. Both PRM-A and PRM-S bind HIV-1 gp120 with a dissociation constant (K_D_) of ~ 0.4 μM and hence exhibit promising antiviral properties [12]. Here, we demonstrate that pradimicins bind primarily to *N*-glycans of the trypanosome surface glycoproteins. Moreover while PRM-A/S is able to bind VSGs, affinity is strongly dependent on *N*-glycan structures and was markedly reduced in PRM-A-resistant parasites. Moreover, binding competition experiments with HHA and CV-N indicate clearly that pradimicins compete with these lectins in the interaction with VSGs. On the other hand, direct evidence for efficient pradimicin binding was provided by SPR analysis. Indeed, VSGs from resistant parasites exhibit a lower capacity to bind pradimicin than parental VSGs further confirming changes in glycan composition in order to overcome the anti-parasitic CBA suppressive effects. Definitive evidence for changes in VSG glycosylation was obtained by analysis of free and procainamide labelled glycans by mass spectrometry and liquid chromatography. Mutant resistant cells exhibited a significant reduction in the proportion of oligomannose type glycans, namely H8N2 and H9N2, while hybrid and complex species accounted for 47% of total glycans *versus* 23% in the parental cell line. In summary, studies on VSG endoglycosidase treatment, lectin binding, lectin blotting and glycan composition show that parasites overcome PRM-A pressure by an altered *N*-glycan processing leading to an enrichment in hybrid and complex *N*-glycan structures presenting lower numbers of α(1,2)-mannose residues prone to bind this CBA.

We sought to establish how changes in glycan composition occur in pradimicin-resistant parasites. It is well-known that VSG glycosylation is accomplished in a site-specific manner by the action of two catalytic OSTs: STT3A activity transfers Man_5_GlcNAc_2_-PP-Dol to asparagines flanked by an acidic sequence yielding paucimannose structures, and STT3B relocates Man_9_GlcNAc_2_-PP-Dol to any remaining asparagine rendering oligomannose *N*-glycans, respectively [19, 27]. These two enzymes were obvious candidates to be responsible for the resistance phenotype however there was a possibility that increased trimming of glycans by α(1,2)-mannosidases is involved. In resistant cells we identified a down-regulation of *TbSTT3A* and *TbSTT3B* mRNA levels that prompts hypoglycosylation and changes in the *N*-glycan nature directed towards a reduction of oligomannose and an increase in paucimannose structures. These modifications clearly minimize their accessibility and the ability for binding pradimicins, and consequently are responsible for the appearance of resistance. In this process TbSTT3B appears to be a major player since overexpression of the enzyme in resistant parasites reversed resistance to pradimicins while conversely RNAi mediated depletion in wild type parasites resulted in high levels of resistance. Thus down-regulation of TbSTTB appears to be the main mechanism involved in pradimicin resistance. A question that remains to be addressed is how long term down-regulation of *TbSTT3A* and *TbSTT3B* mRNA is achieved. It is well-established that trypanosomatids lack the ability to regulate RNA-polymerase II transcription initiation, and the control of mRNA abundance and protein profiles depend largely on RNA-binding proteins [36]. For example, depletion of DRBD3, an RNA binding protein involved in mRNA stability, leads to destabilization of several transcripts and splicing defects, binding preferentially within the 3′-UTR of its target genes, although binding sites within the ORFs and the 5′-UTR are possible [37]. We have explored changes in the sequences of 3’ and 5’-UTRs as well as in the ORFs of *STT3* genes as potentially responsible of *STT3* mRNA down-regulation. No modifications were identified in the UTRs yet a series of mutations were found in the ORFs of *STT3A* and *STT3B*. The possibility that mutations within the coding region are related with the modification of mRNA levels has not been established. RNA-binding proteins that bind within the coding region of mRNAs have been described although their major role is modulation of translation [38]. In addition, the impact of these mutations on the catalytic properties of OSTs was not examined. Hence, how the expression and function of *STT3* genes are regulated in mutant cells remains to be understood.

The striking efficacy *in vivo* further demonstrates that we can obtain highly potent and efficient trypanocidal agents by designing compounds that interact with surface glycans. This is an entirely novel concept that warrants further investigation. Indeed, our data revealed that small-size non-peptidic CBA molecules are emerging as a promising strategy for parasite suppression although further studies will be required to improve the pharmacokinetic properties of this kind of compounds and to achieve sufficient central nervous system penetration and efficacy in the late stage of the disease. Pharmacokinetic data with pradimicin derivatives obtained in previous studies have shown that drug levels in brain tissue and cerebrospinal fluid were lower than those measured in other tissues but detectable at concentrations exceeding 1 μg/g after multiple dosing [39].

Here the trypanocidal activity *in vivo* appears to result from direct interaction of the CBA with bloodstream forms since after treatment the morphology of parasites isolated from the blood would suggest a similar mechanism of action to that observed *in vitro*. Interestingly, an important consequence of glycosylation changes was the strong fitness cost observed in mice models. Resistant parasites were either not infective or exhibited a highly attenuated virulence. These findings are in agreement with previous work that has shown that a correct glycosylation of VSG is critical for optimal and efficient host-parasite interaction [5, 6, 28].

In conclusion, pradimicins exhibit a highly cytotoxic activity against bloodstream forms of *T*. *brucei* and render parasitological cure *in vivo* using an acute model of sleeping sickness. By binding to surface glycans, pradimicins lead to defects in cytokinesis resulting in cell lysis. While specific binding to surface VSGs has been demonstrated, interaction with other glycoproteins cannot be ruled out. All this evidence allows us to propose the development of lectin-mimetic agents, such as the non-peptidic pradimicins, as a novel approach for the design of antitrypanosomal agents.

## Materials and Methods

### Trypanosome cultures

*Trypanosoma brucei brucei* single-marker bloodstream forms (BSF) (antigenic type 1.2, MITat 1.2, clone 221a) strain 427, harbouring T7 RNA polymerase and the tetracycline repressor [40] and *Trypanosoma brucei rhodesiense* EATRO3 ETat1.2 TREU164 [41] were used in this study. The parasites were cultured at 37°C and 5% CO_2_ in HMI-9 with 10% (v/v) or 20% fetal bovine serum, respectively.

### Carbohydrate-binding agents

The following non-peptidic mannose-specific CBAs of prokaryotic origin have been used: pradimicin A (PRM-A, *Actinomadura hibisca*) [7]; pradimicin S (PRM-S, *Actinomadura spinosa* strain A A08 51) [42]; pradimicin Fs (PRM-Fs, *Actinomadura spinosa* strain A A08 51 grown in presence of D-serine) [14]; pradimicin FA-1 mono sugar (PRM-FA-1 mono sugar, *Actinomadura hibisca* P157-2 grown in presence of D-serine) [15]; BMS181184 (synthesized by a semisynthetic process or by direct production from D-serine supplemented fermentation of *Actinomadura* sp) [43]; and BMY28864 (synthesized chemically from PRM-A) [17]. The Amaryllis lectin *Hippeastrum* hybrid agglutinin (HHA) [44], stinging nettle lectin (UDA, *Urtica dioica*) [45], broad-leaved helleborine lectin (EHA, *Epipactis helleborine*) [46], snowdrop lectin (GNA, *Galanthus nivalis*) [47], daffodil lectin (NPA, *Narcissus pseudonarcissus*) [48] and cyanovirin-N (CV-N, *Nostoc ellisporum*) [49]. Tomato lectin (TL) and *Erythrina cristagally* lectin (ECL) were obtained from Vector Laboratories, Inc and ConA from Sigma.

### Generation of PRM-A-resistant cell lines

PRM-A, a non-peptidic CBA with α(1,2) mannose specificity, was used to generate resistant cell lines of *T*. *brucei* bloodstream forms by exposure to increasing concentrations of compound. The process started with a pradimicin concentration equal to the EC_50_ (3.20 ± 0.04 μg/ml) and then stepwise selection was performed, obtaining several strains at escalating PRM-A concentrations of 3.2, 3.6, 10, 25, 50 and 100 μg/ml. Parasites were exposed to a higher drug concentration when the generation time (6–8 hours) had equalled that of the parental line, a process which took around 15–25 days. Resistance stability was checked at 1, 2 or 3 months on PRM-A50 and PRM-A100 strains after removal of the drug pressure.

### Sequencing and VSG221 expression analysis

The coding sequences for PRM-A50 and PRM-A100 VSG221 were amplified by PCR using cDNA as template, cloned in the pGEM-T vector (Promega) and finally sequenced.

VSG221 expression was evaluated by immunofluorescence using an anti-VSG221 polyclonal antibody on PRM-A-resistant cells as described [5]. Briefly, parasites were fixed for 20 min on poly-L-lysine-coated slides with 4% *p-*formaldehyde, washed twice (PBS and 0.2% Tween 20) and blocked during 30 min with Blocking Reagent 1% (Roche). Subsequently, samples were incubated with anti-VSG221 polyclonal antibody for 1 h, labelled with FITC-conjugated anti-rabbit secondary antibody for 1 h, washing before and after labelling. Slides were then dehydrated in methanol for 1 min and finally stained and mounted with Vectashield-DAPI (Vector Laboratories, Inc.). The microscopy and digital image acquisition were performed using a Zeiss Axiophot microscope (Carl Zeiss, Inc.)

### RNA extraction, cDNA synthesis and real time quantitative PCR (RT-qPCR)

Total RNA of parental and PRM-A-resistant cell lines was extracted using TRIzol reagent (Invitrogen), and treated with DNase to avoid a genomic DNA contamination using the RNeasy Micro kit (Qiagen). cDNA was obtained by reverse transcription using iScript cDNA synthesis kit (Bio-Rad). Quantitative PCR assays were carried out in an iCycler IQ real-time PCR detection system (Bio-Rad) using SsoFast EvaGreen Supermix (Bio-Rad). All procedures were performed according to the manufacturer’s instructions. Relative expression of the *TbSTT3A*, *TbSTT3B* and *TbSTT3C* genes was measured as described [5] using the *myosin 1B* gene (Tb927.11.16310) as reference, which was kindly provided by Dr. Navarro [50]. Three independent experiments and sample triplicates were performed in all RT-qPCR assays.

### Small-scale sVSG (soluble-form VSG) isolation and endoglycosidase digestion

The sVSG isolation of PRM-A-resistant strains was performed following the protocol described by Cross *et al* [20, 51] with slight modifications. Pellets from 2 x 10^8^ cells were lysed in 300 μl of hypotonic lysis buffer (10 mM sodium phosphate buffer, pH 8.0 plus protease inhibitor cocktail (Roche)) for 5 min at 37°C. The supernatant containing VSG was collected by centrifugation at 14,000 x *g* for 5 min, loaded onto 0.2 ml of a DE52 (Whatman) and eluated with 10 mM sodium phosphate buffer, pH 8.0. Finally VSG was diluted in water after concentrating and diafiltering on a Nanosep 10K Omega (Pall Corporation).

sVSGs isolated of parental and PRM-A-resistant cell lines were subjected to endoglycosidase digestion. For each enzyme digestion, 1 μg of sVSG was denatured in 10 μl of 0.5% SDS and 0.1 M dithiothreitol for 10 min at 100°C, followed by overnight treatment at 37°C with 500 units of Endo H or PNGase F (New England Biolabs) in the corresponding buffer supplied by the manufacturer.

### Immunofluorescence analysis of VSG expression

To assess the VSG nature in PRM-A-resistant cells, an immunofluorescence analysis using an anti-TbVSG221 polyclonal antibody was carried out. Trypanosomes were fixed in 4% *p-*formaldehyde on poly-L-lysine-coated slides at RT for 20 min, washed (PBS and 0.2% Tween 20) and blocked with Blocking Reagent 1% (Roche) for 30 min. Then, samples were incubated with anti-TbVSG221 for 1 h, washed, probed with FITC-conjugated anti-rabbit antibody for 1 h and washed again. Slides were finally stained and mounted with Vectashield-DAPI (Vector Laboratories, Inc.) after dehydrating in methanol. The microscopy and digital image acquisition were carried out with a Zeiss Axiophot microscope (Carl Zeiss, Inc.)

### Surface plasmon resonance (SPR) analysis

Binding of PRM-A and PRM-S to VSG221 expressed in parental or PRM-A-resistant cell lines was evaluated using SPR on a Biacore T200 instrument (GE Healthcare, Uppsala, Sweden). TbBSF VSG221 was covalently immobilized on a CM5 sensor chip in 10 mM sodium acetate, pH 5, using standard amine coupling chemistry, resulting in chip densities of 826 (low-density) and 6,500 (high-density) RU. The same coupling chemistry was used to immobilize 6,070 RU of PRM-A100-resistant VSG221 to the sensorchip. Interaction studies with PRM-A were performed at 25°C in HBS-P (10 mM HEPES, 150 mM NaCl and 0.05% surfactant P20, pH 7.4) containing 5% DMSO and 10 mM CaCl_2_. Interaction studies with PRM-S were performed in the same buffer without DMSO (due to a markedly higher solubility of PRM-S *versus* PRM-A). A reference flow cell was used as a control for non-specific binding and refractive index changes. Several buffer blanks were used for double referencing. A variety of PRM-A and PRM-S concentrations were injected for 2 min at a flow rate of 30 μl/min and followed by a dissociation phase of 5 min. The CM5 sensor chip surface was regenerated with a single injection of 10 mM NaOH.

### HHA-FITC and CV-N-FITC labelling in the presence of PRM-S

An HHA-FITC conjugate binds to VSG *N*-glycans containing α(1,3) and/or α(1,6) mannose forming a VSG-HHA complex which is rapidly endocytosed [5]. A CV-N-FITC conjugate would bind to glycans containing α(1,2) mannose. Competition experiments with HHA-FITC and CV-N-FITC were used to evaluate binding of PRM-S to VSGs. For this purpose, live parental *T*. *brucei* cells (1.5 x 10^6^ parasites) were washed once with Voorheis PBS (PBS containing 10 mM glucose and 79 mM sucrose), resuspended in 1 ml of serum-free HMI-9 medium containing 1% BSA and preincubated for 20 min at 37°C. Samples were incubated with HHA-FITC (1 μg/ml) or CV-N-FITC (0.6 μg/ml) in the presence or absence of PRM-S, washed twice with cold PBS and resuspended finally in PBS. FACS analysis was carried out using a Becton Dickinson FACSCalibur and BD CellQuest Pro version 4.0.2 software. For microscopy analysis, cells were fixed after labelling with 2% *p*-formaldehyde for 1 h at 4°C, washed, adhered on poly-L-lysine coated slides, dehydrated in methanol and stained with Vectashield-DAPI (Vector Laboratories, Inc.). Vertical stacks of 10–15 slices (0.2 μm steps) were captured using an Olympus microscope and Cell R IX81 software. Deconvolution and pseudo-colouring of images was performed using Huygens Essential software (version 3.3; Scientific Volume Imaging) and Image J software (version 1.37; National Institutes of Health), respectively.

### Labelling assays

UDA-FITC conjugates that bind to *N*-acetylglucosamine residues of VSG *N*-glycans [6], CV-N-FITC and HHA-FITC were used to establish changes in glycan nature. Samples of live parental and resistant *T*. *brucei* cells were prepared and labelled using CV-N-FITC (0.6 μg/ml), HHA-FITC (1 μg/ml) and UDA-FITC (5 μg/ml).

### Uptake assays

Endocytosis dynamics in the parental line upon PRM-S supplementation and in the presence or absence of HHA (1 μg/ml) was determined by uptake of Alexa Fluor 488-dextran 10,000 conjugates (1 mg/ml) in 50 μl of final sample volume. To analyse endocytosis in PRM-A-resistant cell lines, AlexaFluor 594-ConA (100 μg/ml), FITC-transferrin (50 μg/ml), and Alexa Fluor 594-dextran 10,000 (1 mg/ml) conjugates were used as described [5]. All conjugates were purchased from Molecular Probes Inc (Life technologies, Thermo Fisher Scientific Inc). Samples were prepared and analysed as described above for FACS analysis.

### Lectin blotting

In order to study the glycosylation profile of the PRM-A-resistant strains, lectin blotting using TL, ECL and ConA was performed. Both, sVSG (2 μg) and cell pellets (1 x 10^−6^ cell equivalents/sample) coming from hypotonic lysis of PRM-A100 cells were denatured in SDS-sample buffer containing 8 M urea and 50 mM DTT, analysed using MOPS electrophoresis on NuPAGE Bis-Tris 4–12% gradient gel (Invitrogen) and transferred to a nitrocellulose membrane. Proteins were stained with Ponceau S (Sigma) as loading control and blocked with 3% BSA in PBS, previously probing with either biotinylated TL (0.33 μg/ml, Vector Laboratories, Inc.) in a solution containing 50 mM Tris-HCl pH 7.4, 0.5 M NaCl, 0.05% IGEPAL and 0.25% BSA, biotinylated ECL (1 μg/ml, Vector Laboratories, Inc.) in a solution containing 10 mM HEPES pH 7.4, 0.15 M NaCl, 1 mM CaCl_2_, 0.05% IGEPAL and 0.25% BSA, or biotinylated ConA (0.05 μg/ml, Sigma) in PBS containing 1 mM MgCl_2_, 1 mM CaCl_2_, 1 mM MnCl_2_, 0.05% IGEPAL and 0.25% BSA. In all cases, specific inhibitors of lectin binding such as chitin hydrolysate (1:10 dilution, Vector Laboratories, Inc.) for TL, D-lactose (0.2 M, Sigma) for ECL and methyl α-D- mannopyranoside (0.5 M, Sigma) for ConA were used as carbohydrate-specific binding controls. Finally, glycoproteins were detected with Extravidin-peroxidase conjugated (Sigma) by chemiluminescent detection ECL Western Blotting Detection Reagents (GE Healthcare).

### *T*. *brucei* VSG221 *N*-glycan analysis

Glycan profiling by mass spectrometry and liquid chromatography was performed to gain a more detailed view of VSG glycosylation including the relative quantification of glycan distribution. *T*. *brucei* VSG221 samples (50 μg) of parental (*Tb* BSF) and resistant (PRM-A100) cells were denatured and enzymatically deglycosylated with PNGase F according to the manufacturer´s instructions (New England Biolabs, PNGaseF glycerol free). After deglycosylation, the protein fraction was removed by filtration on a 10 kDa spin filter (Amicon Ultra-0.5 ml Centrifugal Filters, Merck Millipore), recovering the free glycans in the filtrate. The released glycans from 50 μg of VSG221 protein were purified over C18 loaded Zip-Tips to remove polymers, the sample was treated with 1 μl of sodium citrate tribasic (1 mM) to favour the exclusive production of sodium adducts and subjected to MALDI-TOF MS analysis. Super-DHB was used as MALDI matrix as a 20 mg/ml solution in ACN. 1 μl of both sample and matrix solution were spotted to the MALDI sample plate into the same spot. The spectra were recorded in reflector positive mode in the 700–3000 Da range, on an UltrafleXtreme III MALDI-TOF, Bruker Daltonics, Germany. Further information on glycan structure was obtained by fragmentation by MALDI-TOF MS selecting the ions 2393 m/z, 2028 m/z and 1663 m/z, and employing in-source fragmentation routine (LIFT, Bruker). Glycan fragments were identified using the Flexcontrol Ultraflex TOF/TOF software application and GlycoWorkBench 2.1 free software.

A relative quantification of the glycan profile was performed by analysing procainamide labelled glycans by UPLC-FLD-MS. Glycans were enzymatically released from 50 μg of filtered VSG221, dried and labelled with procainamide (procainamide/cyanoborohydride in DMSO/AcOH) for 3h at 60°C. The labelled glycans were dried, redissolved in ACN, and analysed by UPLC-FLD on an Acquity UPLC Glycan BEH amide column (1.7 μm, 2.1 mm x 150 mm) in ammonium formate/ACN. Fluorescent labelled glycans were detected with a FLD and an ESI-TOF mass analyser.

### *In vivo* studies

PRM-S, a more soluble analogue of PRM-A, BMS181184 and BMY28864, were used to evaluate the trypanocidal activity *in vivo* of CBAs. Groups containing between three to six C57BL/6 or Balb/C mice (6–8 weeks old) (Jackson Laboratories, Bar Harbor, ME) were infected intraperitoneally with 5 x 10^3^ monomorphic *T*. *brucei brucei* (PRM-S treatment) or 1 x 10^4^ *T*. *brucei rhodesiense* parasites (PRM-S, BMS181184 and BMY28864 treatment), respectively. Different dosages of 25 mg/kg and 50 mg/kg of PRM-S (four dosages) and 50 mg/kg of BMS181184 and BMY28864 (three dosages), were administered once a day intraperitoneally (0.2 ml) starting on the third day post-infection. Compounds were initially dissolved in PBS at 12.5 mg/ml, followed by dilution in 0.5% w/v hydroxypropylmethylcellulose, 0.4% v/v Tween 80 and 0.5% v/v benzyl alcohol used as dosage formulation. One group of mice in each case was also infected and treated with the vehicle as control. Parasitaemia was monitored daily in a haematocytometer and morphology was examined under a microscope from the third day post-infection and after tail blood extraction.

To study the effect of the glycosylation changes observed in PRM-A-resistant parasites on infectivity, 600 monomorphic *T*. *brucei brucei* parasites of parental, PRM-A25, PRM-A50 and PRM-A100 strains were used to infect intraperitoneally between four and seven female C57BL/6 mice per group, respectively (6–8 weeks old). Parasitaemia was monitored daily in a haematocytometer under a microscope from the fourth day post-infection and after tail blood extraction.

### Ethics statement

The animal research described in this manuscript complied with Spanish (Ley 32/2007) and European Union Legislation (2010/ 63/UE). The protocols used were denoted as 1646/13 (PRM-A100 infection), 511/15.2 (PRM-A25 and PRM-A50 infection), 1947/13.A.1, 1947/13.B.1 and DGP.2/2014/CEEA (PRM-S treatment) and 2738/13.A.1 (BMS181184 and BMY28864 treatment) and approved by the Animal Care Committee of the Instituto de Parasitología y Biomedicina “López-Neyra”, CSIC.

### Statistical analysis

We expressed the results as the mean ± SD for each group, and comparisons between groups were performed using Student’s *t*-tests using a commercially available, computer-based statistical package (GraphPad Software Inc.) for all calculations. A *p* value ≤0.05 was considered statistically significant.

## Supporting Information

S1 Fig EC_50_ determination. Growth inhibition profile of *T*. *brucei brucei* single-marker strain 427 bloodstream forms treated with different pradimicin derivatives. Cells were cultured in triplicate for 48 hours with varying concentrations of pradimicins.
(TIF)

S2 Fig Alignment of the VSG221 sequences from parental and PRM-A-resistant cell lines. The alignment was obtained with ClustalW2 (EMBL-EBI).
(TIF)

S3 Fig MALDI-TOF spectra of VSG221 *N*-glycans of parental (Tb *BSF*) and PRM-A100-resistant cell lines. (TIF)

S4 Fig MALDI-TOF fragmentation spectra (LIFT) of H7N6 glycans of the PRM-A100-resistant cell line. (TIF)

S5 Fig MALDI-TOF fragmentation spectra (LIFT) of H6N5 glycans of the PRM-A100-resistant cell line. (TIF)

S6 Fig MALDI-TOF fragmentation spectra (LIFT) of H5N4 glycans of the PRM-A100-resistant cell line. (TIF)

S7 Fig PCR analysis of the *TbSTT3* genes using genomic DNA as template. (A) Scheme of *TbSTT3* genes and the likely rearrangements between *TbSTT3B* and *TbSTT3C* genes, which were observed in an HHA20-resistant cell line and reported previously [5]. Name of the primers used in the study are included in the scheme. (B) PCR product analysis of the full-length and chimeric *TbSTT3* genes according to the scheme shown in panel A in both PRM-A50 and PRM-A100 resistant strains.
(TIF)

S8 Fig Sequencing of the ORFs and UTRs of the *TbSTT3* genes. Scheme of the DNA region containing the *TbSTT3* genes. Sequencing was performed on DNA purified from agarose gels after restriction endonuclease digestion using different primers.
(TIF)

S9 Fig BMY28864 and BMS181184-treatment of Balb/C mice infected with *T*. *brucei rhodesiense* EATRO3 ETat1.2 TREU164. (A and B) Parasitaemia of animals treated with 50 mg/kg of BMY28864 (A) BMS181184 (B) and the vehicle used as control. (C) Kaplan-Meier survival analysis of mice infected and treated. † denotes the day of infection when mice died.
(TIF)

S1 Table Oligosaccharyltransferase amino acid changes encoded by the *TbSTT3A*, *TbSTT3B* and *TbSTT3C* genes in PRM-A100-resistant cells compared to the parental cell line. (DOCX)

S1 Text Text describes the methodology used to perform the plasmid constructs and transfection, as well as the DNA sequencing strategy for oligosaccharyltransferase genes. (DOCX)
