# Supplementary figures and images for: Carbohydrate-Binding Non-Peptidic Pradimicins for the Treatment of Acute Sleeping Sickness in Murine Models
Source: PLoS Pathog. 2016 Sep 23;12(9):e1005851. doi: 10.1371/journal.ppat.1005851 (PMC5035034; doi:10.1371/journal.ppat.1005851)

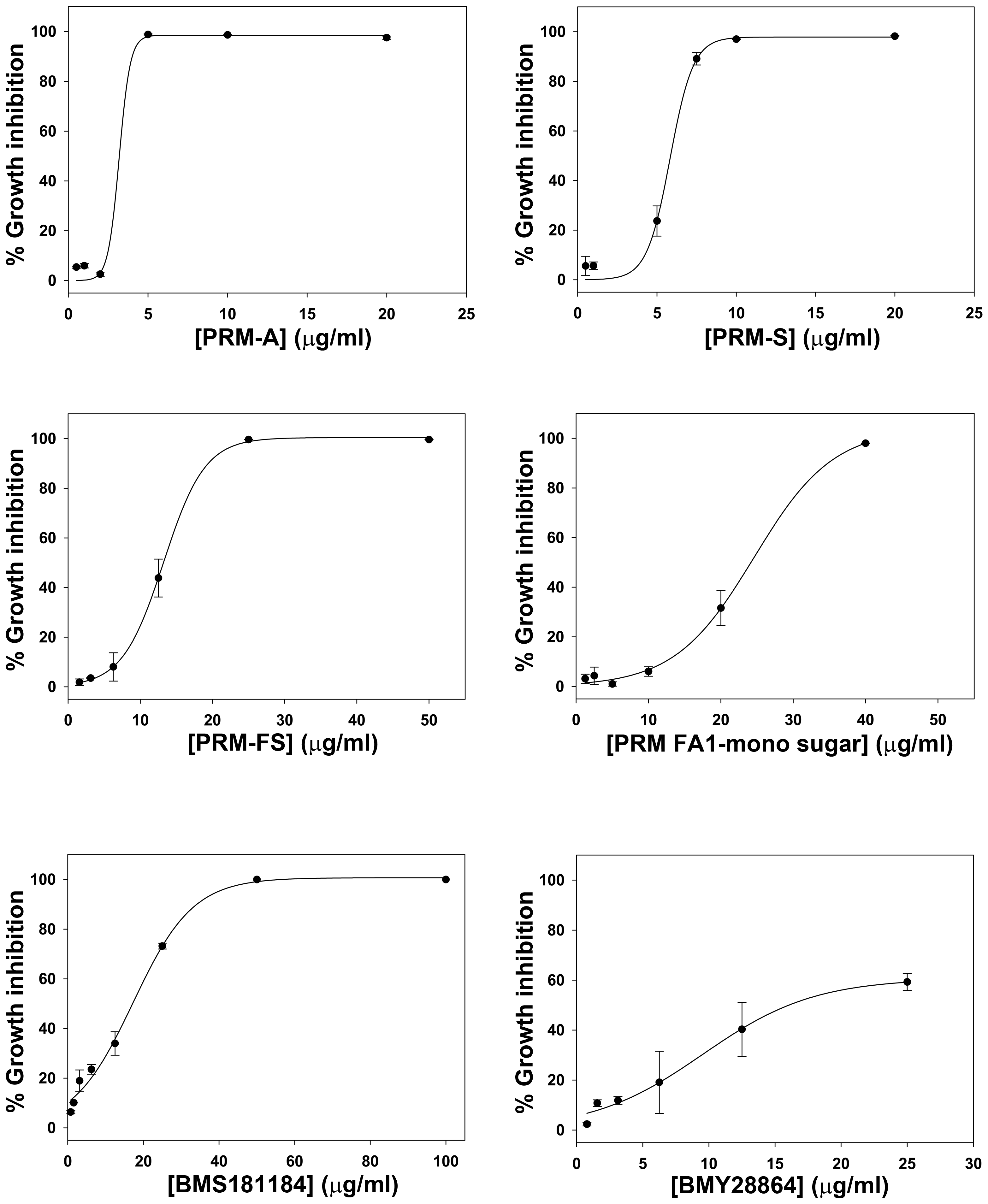

Supplement: S1 Fig — Growth inhibition profile of T. brucei brucei single-marker strain 427 bloodstream forms treated with different pradimicin derivatives. Cells were cultured in triplicate for 48 hours with varying concentrations of pradimicins. (TIF) [file ppat.1005851.s001.tif]

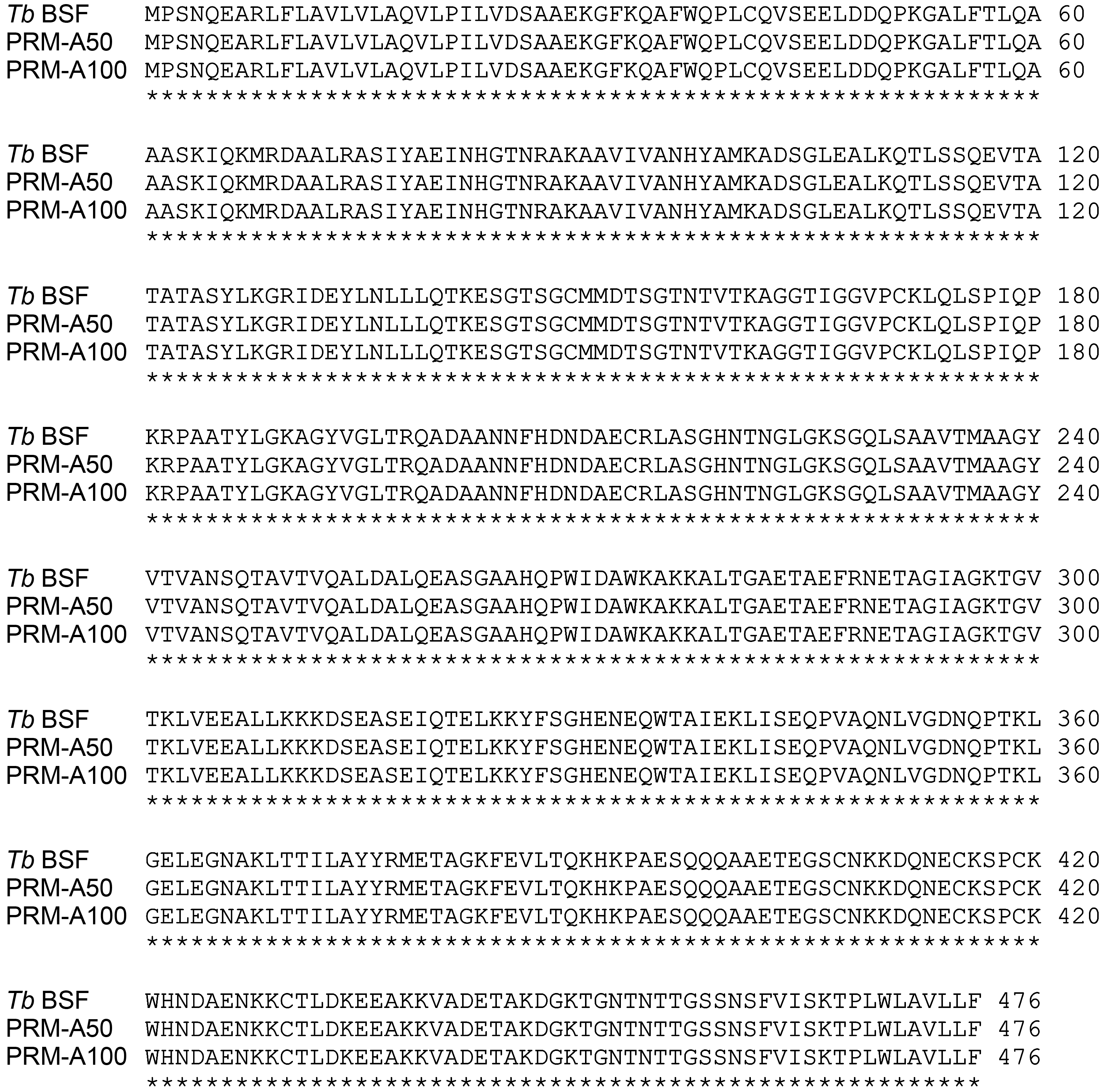

Supplement: S2 Fig — The alignment was obtained with ClustalW2 (EMBL-EBI). (TIF) [file ppat.1005851.s002.tif]

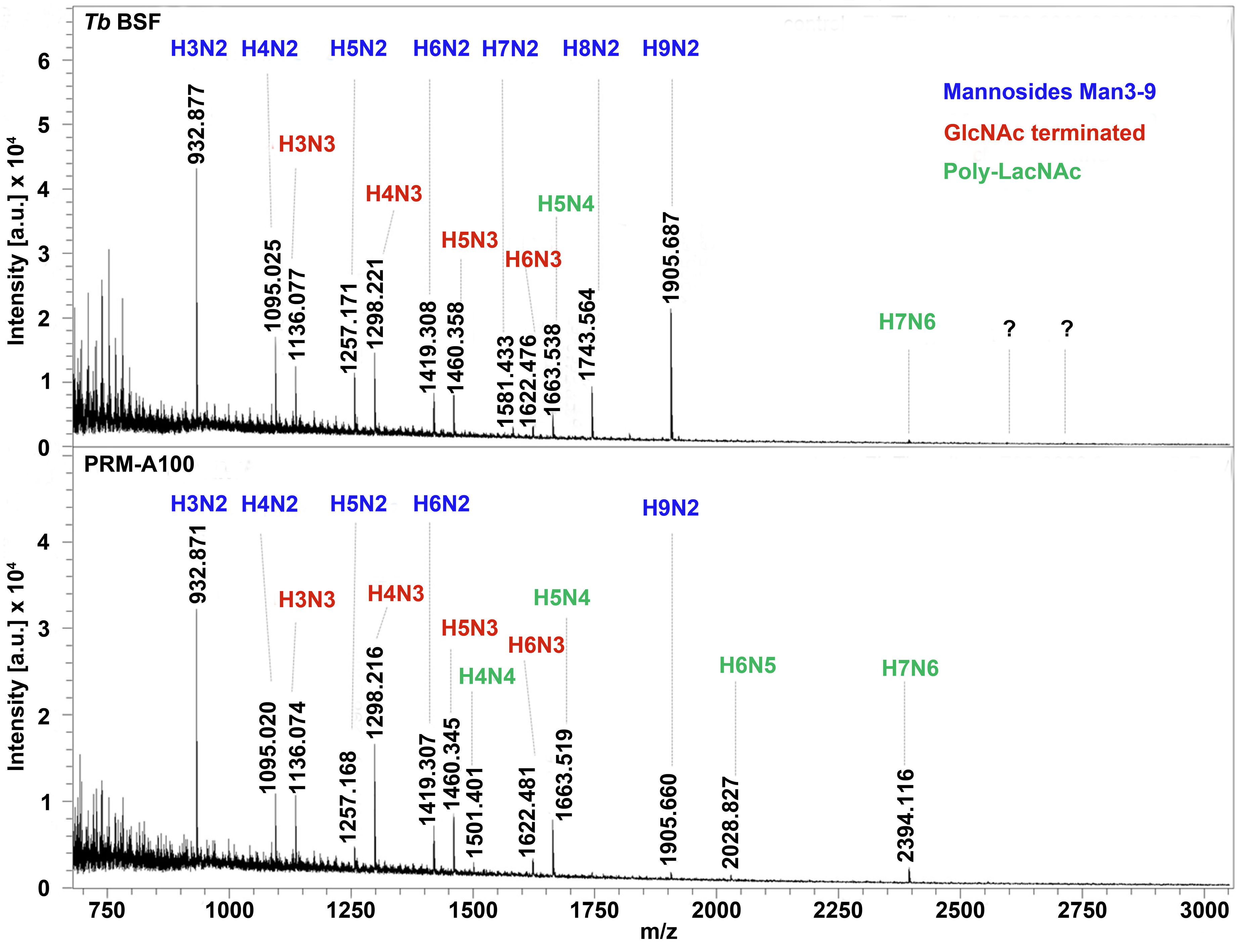

Supplement: S3 Fig — (TIF) [file ppat.1005851.s003.tif]

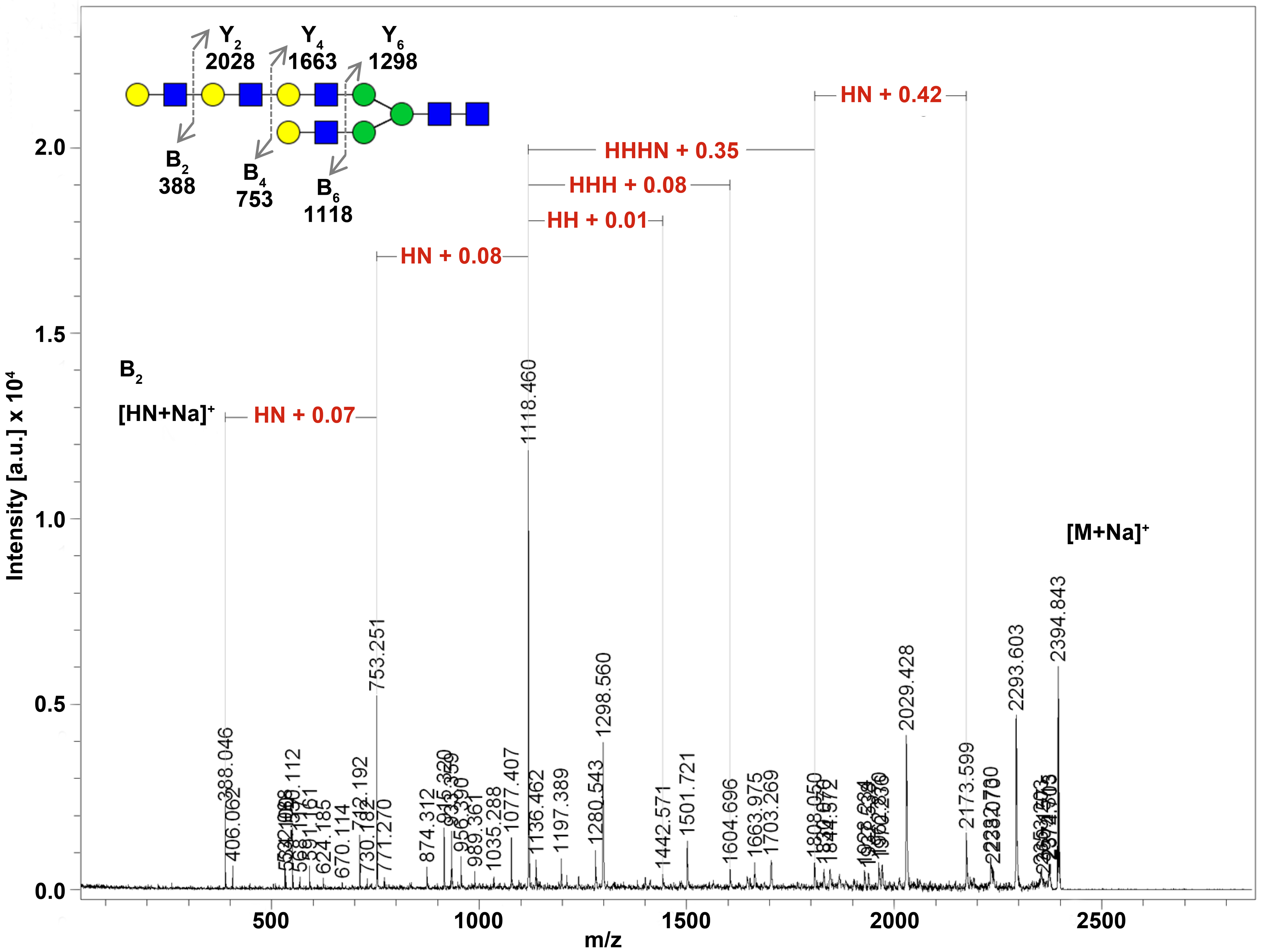

Supplement: S4 Fig — (TIF) [file ppat.1005851.s004.tif]

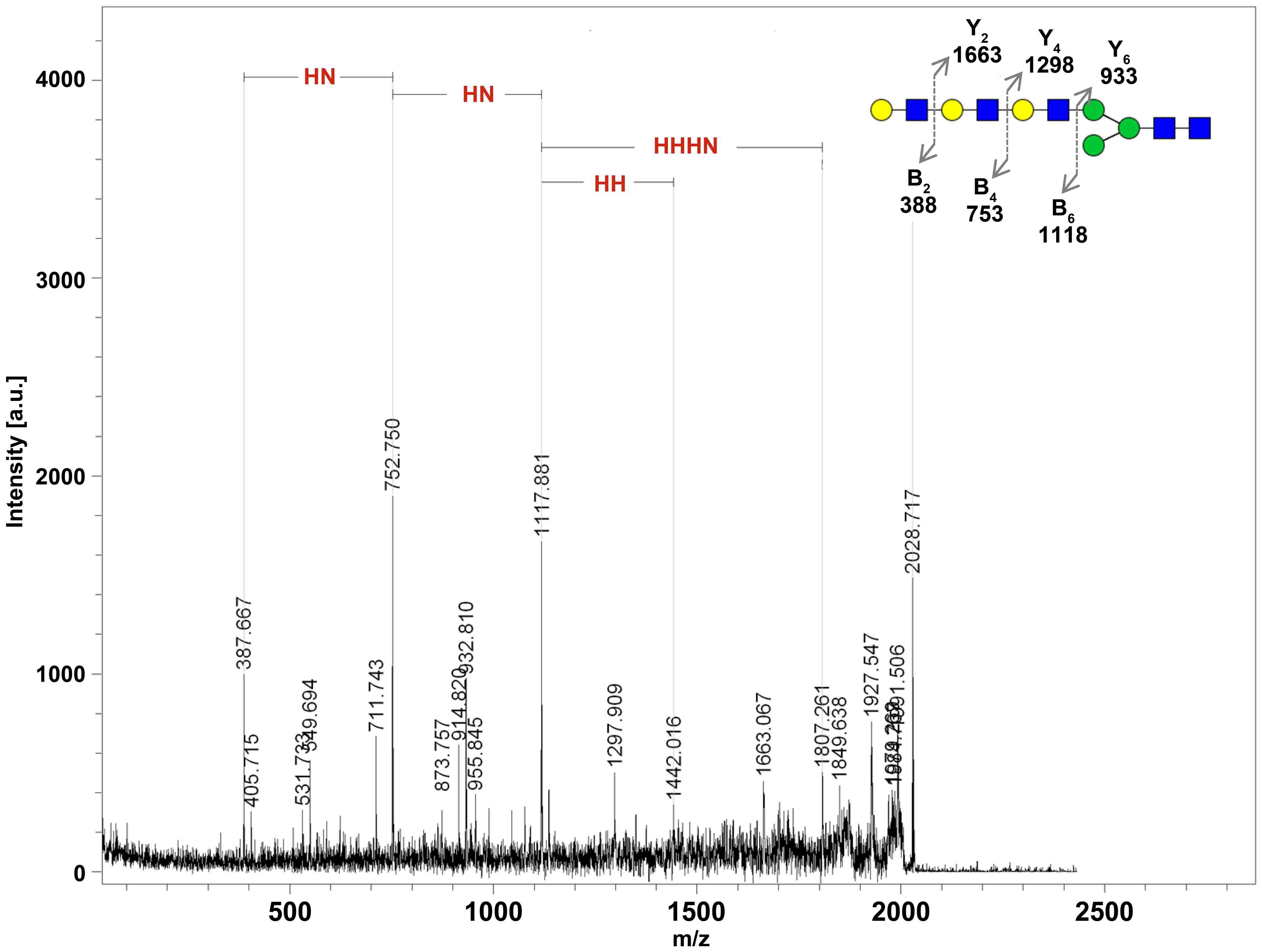

Supplement: S5 Fig — (TIF) [file ppat.1005851.s005.tif]

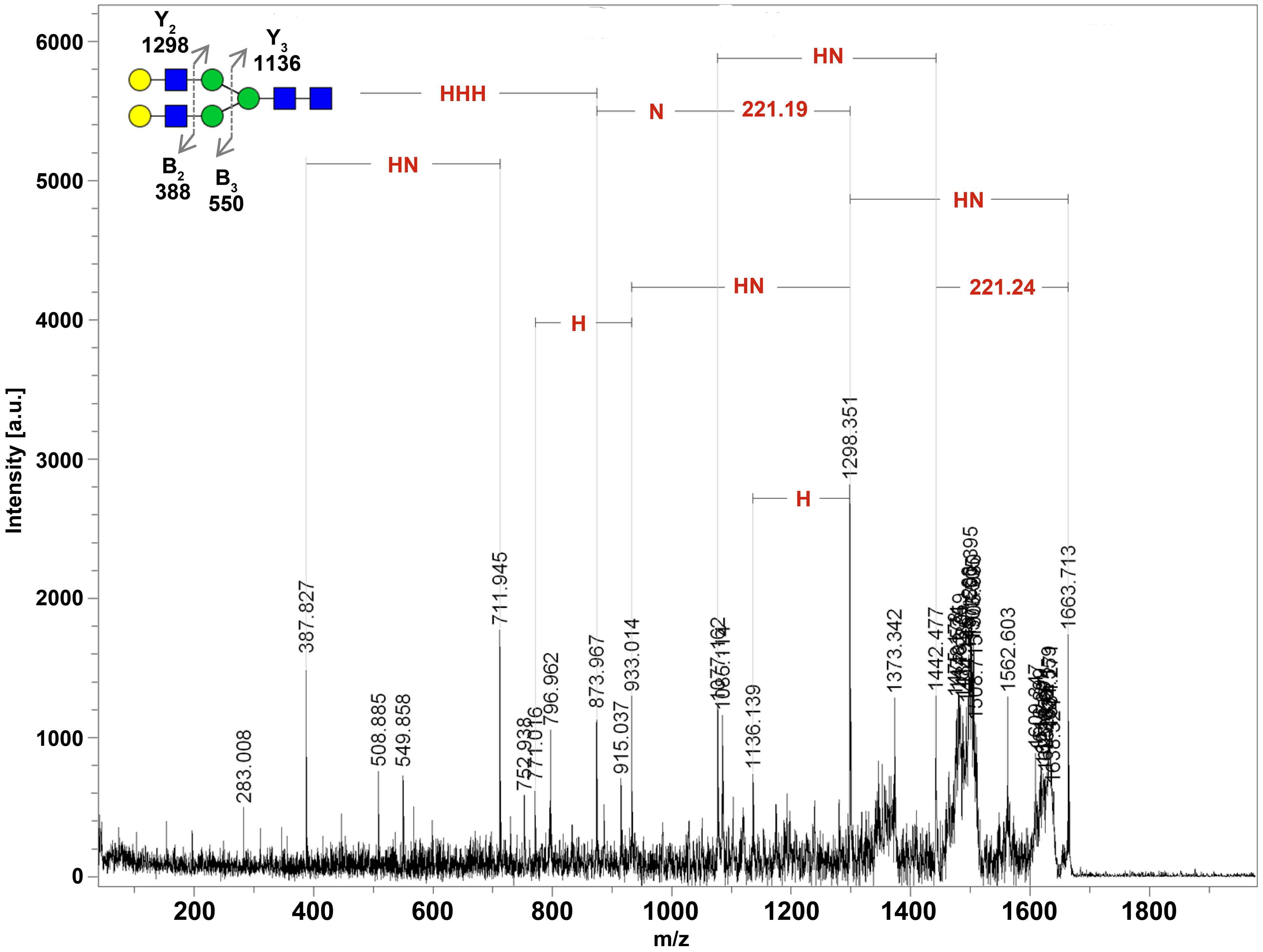

Supplement: S6 Fig — (TIF) [file ppat.1005851.s006.tif]

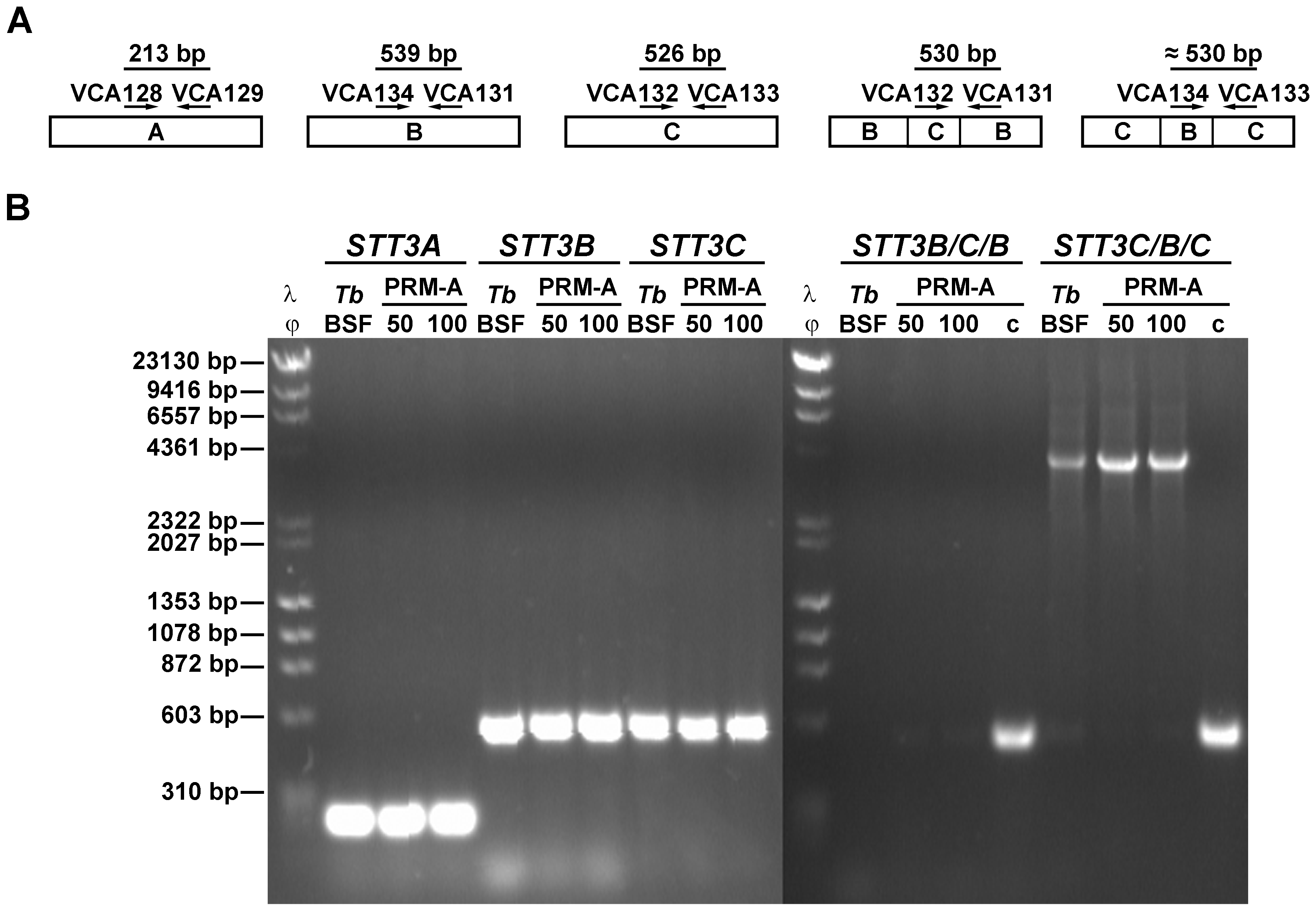

Supplement: S7 Fig — (A) Scheme of TbSTT3 genes and the likely rearrangements between TbSTT3B and TbSTT3C genes, which were observed in an HHA20-resistant cell line and reported previously [5]. Name of the primers used in the study are included in the scheme. (B) PCR product analysis of the full-length and chimeric TbSTT3 genes according to the scheme shown in panel A in both PRM-A50 and PRM-A100 resistant strains. (TIF) [file ppat.1005851.s007.tif]

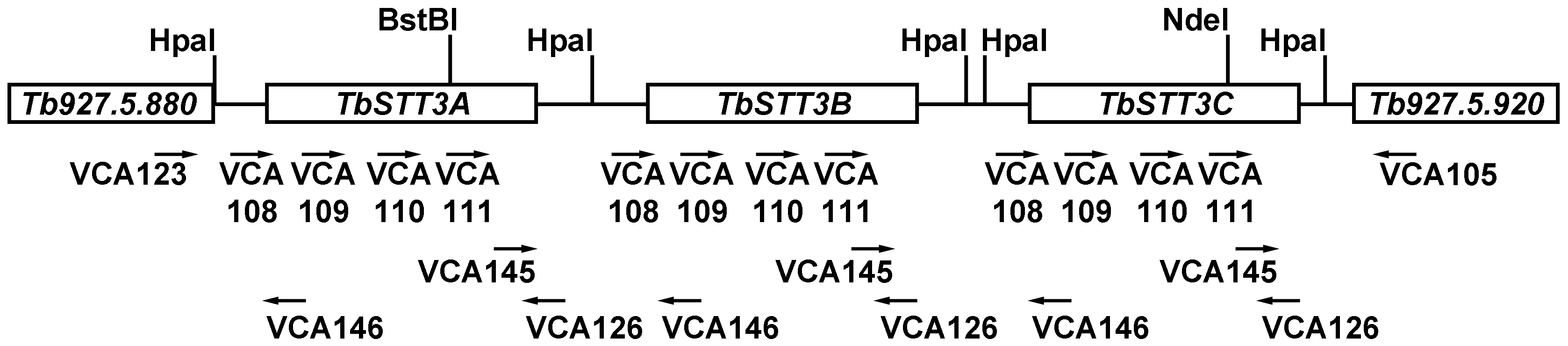

Supplement: S8 Fig — Scheme of the DNA region containing the TbSTT3 genes. Sequencing was performed on DNA purified from agarose gels after restriction endonuclease digestion using different primers. (TIF) [file ppat.1005851.s008.tif]

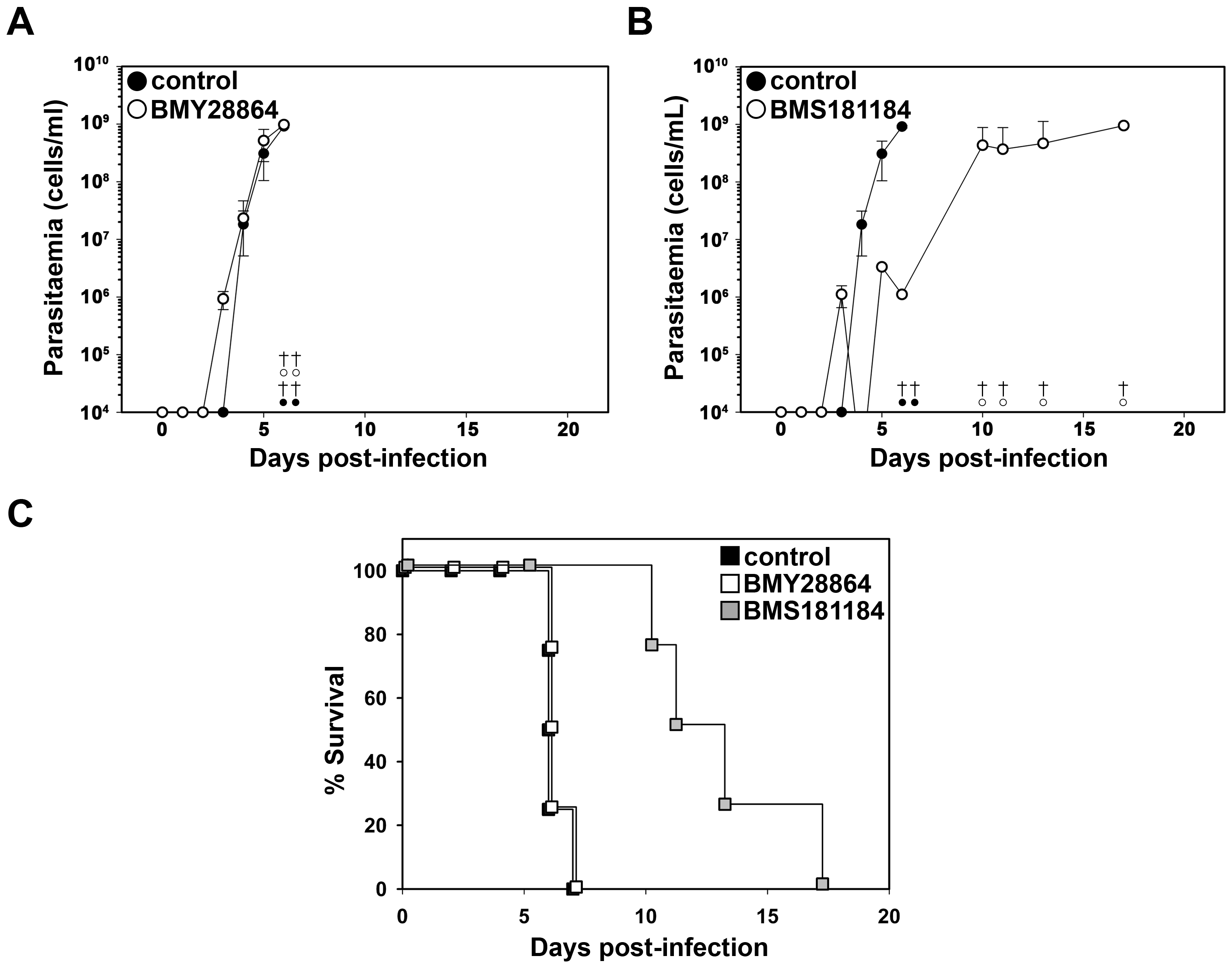

Supplement: S9 Fig — (A and B) Parasitaemia of animals treated with 50 mg/kg of BMY28864 (A) BMS181184 (B) and the vehicle used as control. (C) Kaplan-Meier survival analysis of mice infected and treated. † denotes the day of infection when mice died. (TIF) [file ppat.1005851.s009.tif]
