# Supplementary material for: Carbohydrate-Binding Non-Peptidic Pradimicins for the Treatment of Acute Sleeping Sickness in Murine Models
Source: PLoS Pathog. 2016 Sep 23;12(9):e1005851. doi: 10.1371/journal.ppat.1005851 (PMC5035034; doi:10.1371/journal.ppat.1005851)
Supplement: S1 Table — (DOCX) [file ppat.1005851.s010.docx]

**Table S1. Oligosaccharyltransferase amino acid changes encoded by *TbSTT3A*, *TbSTT3B* and *TbSTT3C* genes in PRM-A100-resistant strains compared to the parental cell line.**

| ***TbSTT3A*** | ***TbSTT3B*** | ***TbSTT3C*** |
| --- | --- | --- |
| E510G |  | No changes |
| K513E | E629K |  |
| L705P |  |  |
