# Supplementary material for: Carbohydrate-Binding Non-Peptidic Pradimicins for the Treatment of Acute Sleeping Sickness in Murine Models
Source: PLoS Pathog. 2016 Sep 23;12(9):e1005851. doi: 10.1371/journal.ppat.1005851 (PMC5035034; doi:10.1371/journal.ppat.1005851)
Supplement: S1 Text — (DOCX) [file ppat.1005851.s011.docx]

**Supporting Information**

## Plasmid constructs and transfection

*TbSTT3A* and *TbSTT3B* genes were silenced by an RNAi strategy individually or in combination. In the case of *STT3B* silencing, the *Tb* BSF *STT3B*-RNAi cell line was previously generated in our laboratory [[1](#_ENREF_1)]. To deplete TbSTT3A, a fragment of 499 bp (*TbSTT3A*) of the coding sequence was amplified by PCR from genomic DNA using PCR Extender Polymerase Mix (5PRIME) and the pair of primers previously described [[2](#_ENREF_2)], but including different restriction sites: 5’- GCG GAT CCA AGC TTC CCT CTT TGG GGG TGC-3’ (BamHI and HindIII restriction sites underlined) and 5’- GTT AAC GGG CCC ATT GGG TAG ATC AGT CAC G -3’ (HpaI and ApaI restriction sites underlined). In the case of STT3A/B depletion in combination by RNAi, the same pair of primers with modifications in the restriction sites was used. Thus, to amplify the *STT3A* fragment the primers 5’- GCG GAT CCA AGC TTC CCT CTT TGG GGG TGC-3’ (BamHI and HindIII restriction sites underlined) and 5’- GCG CTA GCA TTG GGT AGA TCA GTC ACG -3’ (NheI restriction site underlined) were used, whereas to amplify the *STT3B* fragment the primers 5’- GCG CTA GCG ATG ATT TCT TTG GTT ACC-3’ (NheI restriction site underlined) and 5’- GCC ATA TGC TCA GAA TAT ATC CGG AAG -3’ (NdeI restriction site underlined) were used. The PCR products were cloned into the pGEM-T vector in tandem to generate the fragment STT3A/B, which was again amplified with the primers 5’- GCG GAT CCA AGC TTC CCT CTT TGG GGG TGC (BamHI and HindIII restriction sites underlined) and 5’- GTT AAC GGG CCC CTC AGA ATA TAT CCG GAA-3’ (HpaI and ApaI restriction sites underlined). In both cases, the resulting fragments were cloned into pGR19 [[3](#_ENREF_3)] in the HindIII and ApaI sites and subsequently cloned in the opposite orientation using the BamHI and HpaI restriction sites yielding the plasmids pGRV200 (*STT3A*-RNAi) and pGRV202 (*STT3A/B*-RNAi).

In addition, for RNAi of *TbSTT3A*/*B/C*, a fragment of 499 bp of the coding sequences was amplified by PCR from genomic DNA using PCR Extender Polymerase Mix (5PRIME) and the pair of primers previously described [[2](#_ENREF_2)], but including different restriction sites: 5’- GCG GAT CCA AGC TTG TAG CCA TTC ATC GTG TGC -3’ (BamHI and HindIII restriction sites underlined) and 5’- GTT AAC GGG CCC GGT ACG CAA ATC GCA AGG -3’ (HpaI and ApaI restriction sites underlined). The amplified fragment was cloned into pGR19 [[3](#_ENREF_3)] as described above resulting in the plasmid pGRV203 (*STT3A/B/C*-RNAi).

For overexpression studies in the *Tb* BSF PRMA-100 resistant cell line, the *TbSTT3A* ORF was obtained by PCR using the plasmid pGEMTbSTT3A, described previously [[1](#_ENREF_1)], as DNA template and the pair of primers: 5’- GCA AGC TTA TGA CGA AAG GTG GGA AAG TAG -3’ (HindIII restriction site underlined) and 5’- GCG GAT CCT CAT TCG TAA TGG AAC CGC TTC-3’ (BamHI restriction site underlined). The PCR product was cloned into the pMIG75 expression plasmid containing a tetracycline-inducible *parp* promoter and the nourseothricin resistance gene (*nat*) as selectable marker (kindly provided by Dr. Navarro), yielding the plasmid pGRV201. For overexpression of TbSTT3B, the plasmid pGRV137 described previously was used [[1](#_ENREF_1)].

All plasmids were linearized with NotI and used to transfect the parental line or PRMA-100 mutant parasites in order to generate *STT3*-RNAi (pGRV200, pGRV202 and pGRV203) or STT3-overexpressing (pGRV137 and pGRV201) transfectants, respectively, according to the protocol described previously [[4](#_ENREF_4), [5](#_ENREF_5)]. Transfectans were selected with 5 μg/ml of hygromycin, 0.1 μg/ml of puromycin or 30 μg/ml of nourseothricin.

**DNA sequencing strategy of oligosaccharyltransferase genes**

Genomic DNA of parental and PRM-A100 cell lines was extracted from 5 x 10^7^ parasites using DNAzol^®^ reagent (Invitrogen) according to the manufacturer’s instructions.

The three OST genes, *TbSTT3A* (Tb927.5.890), *TbSTT3B* (Tb927.5.900) and *TbSTT3C* (Tb927.5.910) (GeneDB database), are located in a locus of 10489 base pairs. To analyse the open reading frames of the OST gene sequences as well as the corresponding 5’ and 3’ UTR´s, the locus described above was amplified by PCR in two fragments with pairs of gene-specific primers designed from the flanking gene sequences found in the GeneDB database (Tb927.5.880 and Tb927.5.920) and the variable region of the *TbSTT3B* gene. The primer sequences used were: 5’- CGA CCA CCC TGT GAT ACC GTT GAC CC-3’ (VCA123, Tb927.5.880), 5’-GTT CCC AGT GCT GTT AGC GGG CGG TGG AG-3’ (VCA105, Tb927.5.920), 5’- GGG GCT CGA CAT GCT GTC TGC GAA TTG C-3’ (VCA106, *TbSTT3B*) and 5’- CAG ATT ATA ATG AGG GCA ACT GTC GGT GG-3’(VCA107, *TbSTT3B*). The PCR products were sequenced with primers designed to map the whole region after restriction endonuclease digestion (HpaI, BstBI and NdeI) (S5 Fig). Primers used were: 5’- GAG GGA ACC GTG TGT GAG CGA AGT C -3’ (VCA108), 5’- GCG ATC AAT GGC TGG TGA ATT TGA C (VCA109), 5’- GAA ATC CCC TCG TGG ATT CTG TGG CTG -3’ (VCA110), 5’- CGG GTA TCA AAT CAC TGG AAT TGG C (VCA111), 5’-GCG GAT CCC TAA CCC ATC TGA CGC ATA TAC GCA CG -3’ (VCA126), 5’- CAG TAC CCG CCA GCG AAG GAG ATC-3’ (VCA145) and 5’- CCC TCA CCA GCA CCA TCA CTC TG-3’ (VCA146).

**References**

1. Castillo-Acosta VM, Vidal AE, Ruiz-Perez LM, Van Damme EJ, Igarashi Y, Balzarini J, et al. Carbohydrate-binding agents act as potent trypanocidals that elicit modifications in VSG glycosylation and reduced virulence in *Trypanosoma brucei*. Mol Microbiol. 2013. Epub 2013/08/10. doi: 10.1111/mmi.12359. PubMed PMID: 23926900.

2. Izquierdo L, Schulz BL, Rodrigues JA, Guther ML, Procter JB, Barton GJ, et al. Distinct donor and acceptor specificities of *Trypanosoma brucei* oligosaccharyltransferases. EMBO J. 2009;28(17):2650-61. Epub 2009/07/25. doi: emboj2009203 [pii]

10.1038/emboj.2009.203. PubMed PMID: 19629045; PubMed Central PMCID: PMC2722254.

3. Clayton CE, Estevez AM, Hartmann C, Alibu VP, Field M, Horn D. Down-regulating gene expression by RNA interference in *Trypanosoma brucei*. Methods Mol Biol. 2005;309:39-60. Epub 2005/07/02. doi: 1-59259-935-4:039 [pii]

10.1385/1-59259-935-4:039. PubMed PMID: 15990397.

4. Wirtz E, Hoek M, Cross GA. Regulated processive transcription of chromatin by T7 RNA polymerase in *Trypanosoma brucei*. Nucleic Acids Res. 1998;26(20):4626-34. Epub 1998/10/01. doi: gkb742 [pii]. PubMed PMID: 9753730; PubMed Central PMCID: PMC147901.

5. Wirtz E, Leal S, Ochatt C, Cross GA. A tightly regulated inducible expression system for conditional gene knock-outs and dominant-negative genetics in *Trypanosoma brucei*. Mol Biochem Parasitol. 1999;99(1):89-101. Epub 1999/04/24. doi: S016668519900002X [pii]. PubMed PMID: 10215027.
